# Low-Temperature and Room-Temperature Surface-Activated Au–Au Bonding: Surface Requirements, Preparation Methods, and Emerging Applications

**DOI:** 10.3390/s26185939

**Published:** 2026-09-19

**Authors:** Mohammed Al-Mahmodi, Mousa Al-Zanina, Riadh Al-Haidari, Masahito Takakuwa, Michitaka Yamamoto, Mark D. Poliks, Seiichi Takamatsu

**Affiliations:** 1 School of Systems Science and Industrial Engineering, Binghamton University, Binghamton, NY 13902, USA; malmahmodi@binghamton.edu (M.A.-M.); malzanina@binghamton.edu (M.A.-Z.); ralhaid1@binghamton.edu (R.A.-H.); mpoliks@binghamton.edu (M.D.P.); 2 Department of Electrical Engineering and Information Systems, The University of Tokyo, 7-3-1 Hongo, Bunkyo-ku, Tokyo 113-8656, Japan; tsuyukusa-masakuwa@g.ecc.u-tokyo.ac.jp; 3 Department of Precision Engineering, Graduate School of Engineering, The University of Tokyo, Tokyo 113-8656, Japan; yamamoto-michitaka@g.ecc.u-tokyo.ac.jp

**Keywords:** Au–Au direct bonding, surface-activated bonding, thermocompression bonding, wafer-level packaging, surface roughness, heterogeneous integration, flexible hybrid electronics

## Abstract

Gold-to-gold (Au–Au) bonding forms an oxide-resistant metallic interface without solder or conductive adhesive, making it attractive for heterogeneous integration, MEMS sealing, optoelectronic packaging, and flexible hybrid electronics (FHEs). This review focuses on low-temperature and room-temperature surface-activated Au–Au direct bonding and emphasizes the roles of surface roughness and activation state. Compared with thermocompression bonding (TCB), low-temperature and room-temperature bonding impose stricter surface requirements because heat and pressure in TCB can deform asperities and increase real contact area. Room-temperature bonding instead depends strongly on the surface condition before contact. Successful bonding generally requires very smooth Au surfaces, activation, and contamination control. Reported roughness ranges from below 0.5 nm for smooth sputtered or transferred Au films to tens or hundreds of nanometers for rough plated Au before smoothing. Smoothing strategies can therefore expand the direct-bonding process window. Plasma activation removes contaminants and increases surface reactivity; Ar plasma promotes strong bonding, whereas O_2_ plasma can form Au oxide and weaken the interface. Water-vapor plasma-assisted bonding (WVPAB) further enables bonding on rougher electrodes and flexible polymer substrates. Applications include optoelectronic integration, MEMS hermetic packaging, heterogeneous integration, and FHE. Remaining challenges include wafer-scale roughness control, activated-surface lifetime, patterned Au bonding, long-term reliability, and mechanism-based process optimization.

## 1. Introduction

Au is used as a bonding and electrode material due to its chemical inertness, high conductivity, and resistance to oxidation [1]. These characteristics allow direct metallic contact without flux or solder, which enables low-resistance and stable interfaces. These material advantages make Au a suitable bonding interface. At the same time, the integration of dissimilar materials creates a need for low-temperature direct bonding because differences in their coefficients of thermal expansion can generate thermomechanical stress and cracking during high-temperature processing and subsequent cooling. Low-temperature Au–Au direct bonding addresses this limitation by enabling direct electrical and mechanical integration while reducing the thermal load imposed on the bonded materials.

In microelectronics and flexible device fabrication, bonding refers to the process of joining two materials such as thin films, wafers, or device layers, so that they form a stable, electrically and mechanically integrated structure [2]. Depending on the materials and application, bonding can occur through mechanical adhesion, chemical reactions, or atomic diffusion at the interface [3]. For high-performance systems, particularly in three-dimensional integration (3D ICs) and FHE, the goal is often direct (adhesive-free) bonding of two solid surfaces. It is about joining them without intermediate polymer adhesives or solder layers to form a clean, hermetic, and low-resistance interface [4,5].

Within this bonding landscape, Au-based bonding technologies have evolved significantly. Early Au-based bonding in packaging relied on TCB at high temperatures and pressure. A major shift came with surface-activated bonding (SAB), where ion or plasma activation produces atomically clean, highly reactive metal surfaces so that room-temperature bonding becomes feasible. Because TCB is already extensively reviewed in the literature, it is not the focus of this study. Instead, this review examines low-temperature and room-temperature Au–Au direct bonding. The review covers surface roughness control strategies, activation methods, and application demonstrations in optical devices, MEMS packaging, heterogeneous integration, and flexible hybrid electronics.

### 1.1. Direct and Hybrid Bonding

**Figure 1:**
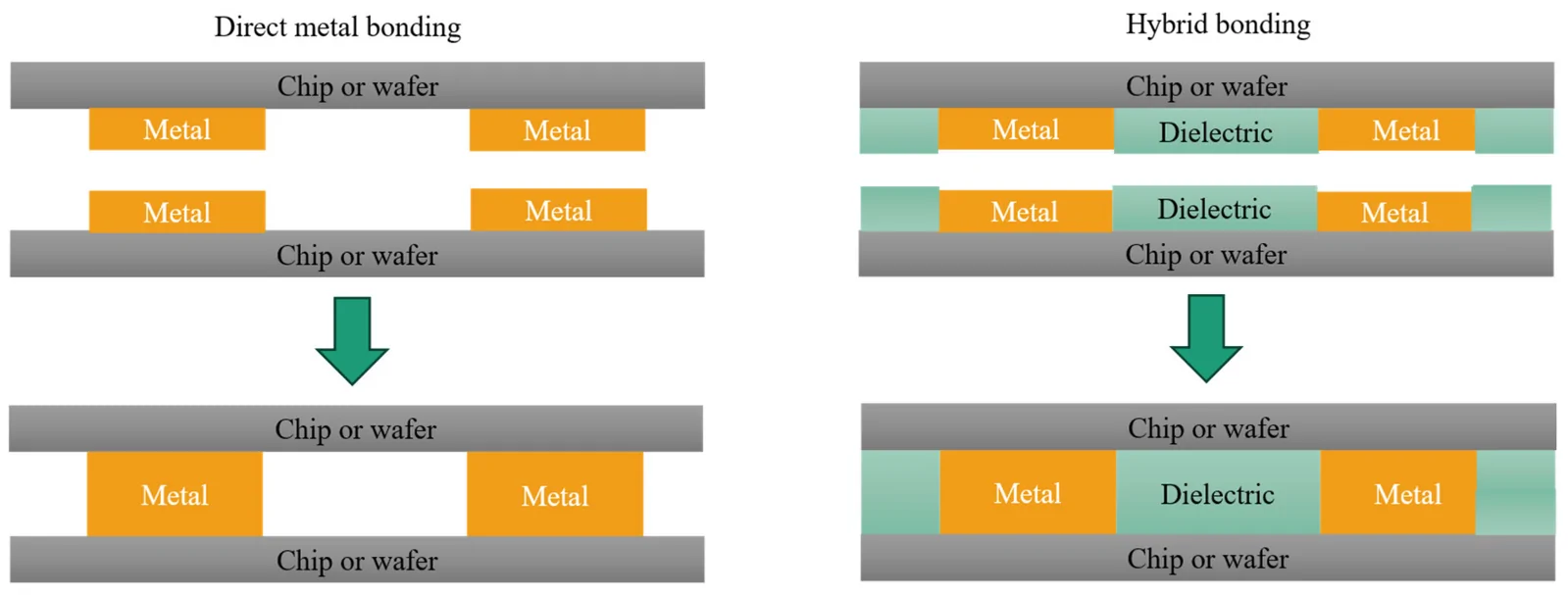
Direct and hybrid bonding concept.

Before examining the Au–Au room/low temperature direct bonding, it is important to distinguish direct bonding from hybrid bonding. Direct bonding is the general concept which refers to joining two surfaces without an intermediate solder or adhesive layer using heat, pressure, or surface activation. In direct bonding, the contact between two atomically clean and smooth surfaces allows interatomic forces, such as van der Waals attraction [3], hydrogen bonding [6], or metallic diffusion [5], to form a continuous interface. When both surfaces are metallic, for instance in Au–Au direct bonding, this leads to electrical conduction with minimal contact resistance [7]. Hybrid bonding is a special case of direct bonding, which refers to the formation of both metallic and dielectric bonds (e.g., Cu/SiO_2_ or Au/SiO_2_ interfaces) [8]. This approach is widely used in wafer-level packaging and 3D integration. The metal pads provide electrical connections, and the surrounding dielectric layers ensure mechanical stability and insulation. It also combines the electrical benefits of direct metal contact with the structural robustness of dielectric adhesion [9,10]. Figure 1 shows a schematic comparison of direct metal bonding and hybrid bonding. Direct metal bonding is dominated by metal-to-metal interfacial contact and typically requires surface cleaning or activation. Those conditions combined with applied normal pressure, and in some cases heat, to achieve intimate contact and diffusion across the interface. Hybrid bonding combines metal-to-metal and dielectric-to-dielectric bonding at the same aligned interface and usually involves planarized surfaces, activation, pre-bonding, and subsequent pressure and/or annealing [11,12]. Table 1 summarizes the key terminology used throughout this review.

### 1.2. Review Scope

This review focuses on Au–Au direct bonding for microelectronics packaging, heterogeneous integration (HI), and FHE. The review examines bonding approaches in which gold is the main bonding interface. It emphasizes the underlying bonding mechanisms, process conditions, surface preparation, interface characterization, and reliability behavior.

The scope is defined by the literature using a Scopus advanced search and Web of Science. It has been built around the terms Au–Au bonding, gold-to-gold bonding, low-temperature bonding, room-temperature bonding, TCB, SAB, plasma-activated bonding, and WVPAB. The application terms include heterogeneous integration, packaging, wafer, chip, die, 3D integration, 3D IC, FHE. Accordingly, this review focuses on surface-activated and surface-engineered Au–Au bonding at low and room temperature. TCB is discussed only as a technical baseline for comparison, while other established Au–Au bonding families, such as atomic-diffusion and ultrasonic bonding, are outside the detailed scope of this review.

Studies involving wafer-to-wafer, chip-to-wafer, die-level, and flexible-substrate bonding are included when they provide useful technical insight into Au–Au interface formation, process limitations, or reliability performance. More attention is given to studies that report experimental details on bonding temperature and pressure, surface cleanliness and roughness, activation methods, interface evolution, bonded area quality, and electrical or mechanical reliability.

**Figure 2:**
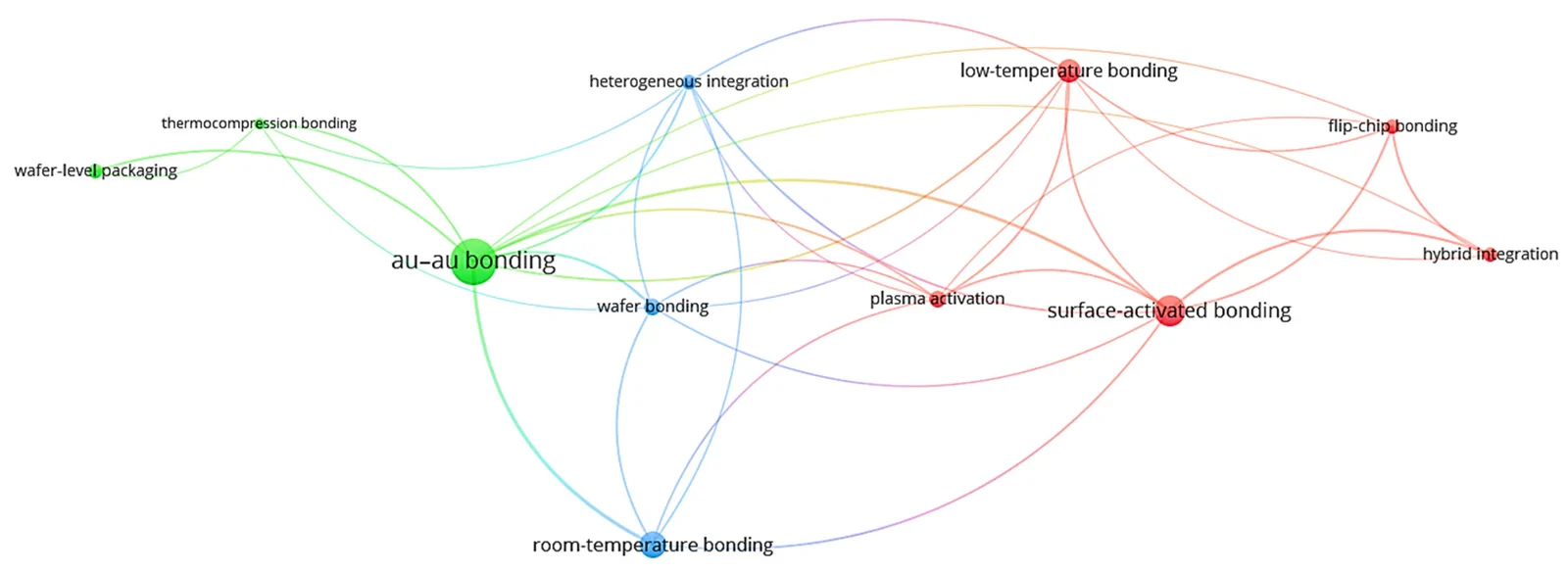
Keyword co-occurrence network of Au–Au bonding research generated using VOSviewer 1.6.20.0. Node size represents keyword frequency, links indicate co-occurrence relationships, and colors denote thematic clusters, last update 17 June 2026.

A keyword co-occurrence analysis was performed using VOSviewer 1.6.20.0, as shown in Figure 2, to identify the main research themes and relationships within the Au–Au bonding literature. Author keywords from the selected publications were analyzed to determine how frequently different terms appear together across studies.

Three main thematic groups can be distinguished in the network. One group links Au–Au bonding, thermocompression bonding, and wafer-level packaging, representing the established use of Au bonding in packaging and thermomechanical joining. A second group connects room-temperature bonding, wafer bonding, and heterogeneous integration, reflecting the transition toward low-thermal-budget wafer and material integration. The third group contains low-temperature bonding, surface-activated bonding, plasma activation, flip-chip bonding, and hybrid integration, showing the close relationship between surface preparation and emerging low-temperature integration routes. The links between these groups indicate that the field has evolved from conventional thermocompression and wafer packaging toward surface-engineered bonding for room-temperature and heterogeneous integration. This bibliometric structure therefore supports the organization of this review, in which TCB is treated as the technical baseline, and surface preparation becomes the central issue as bonding temperature and pressure are reduced.

Existing reviews have addressed low-temperature bonding across multiple material systems, hybrid bonding for three-dimensional integration, and bonding technologies for specific device applications [5,10,12]. The present review differs by concentrating specifically on low-temperature and room-temperature Au–Au direct bonding and organizing the literature around the surface conditions that determine whether direct metallic contact can be achieved. More emphasis is placed on the relationship between surface roughness, surface activation, contamination control, Au-film structure, and bonding conditions. The review also compares low-pressure and atmospheric-pressure activation routes. It also examines strategies for adapting rough electroplated and patterned Au surfaces to low-temperature bonding and links these surface requirements to metrology, reliability, and wafer-scale manufacturability.

### 1.3. Review Methodology

The review was conducted using a structured literature-screening process based on PRISMA principles. Scopus and Web of Science were searched using the strategy summarized in Table 2. The searches identified 191 records, including 91 records from Scopus and 100 records from Web of Science. After DOI- and title-based duplicate removal, 179 unique records remained for screening.

The titles and abstracts of the 179 records were screened according to the predefined scope of the review. Studies were retained when Au constituted the actual bonding interface and when the publication provided information relevant to Au–Au interface formation, bonding conditions, surface preparation, surface morphology, device integration, interface characterization, or reliability. Wafer-to-wafer, chip-to-wafer, die-level, and flexible-substrate studies were considered. Studies were excluded when Au was not the actual bonding interface, when bonding relied primarily on solder or adhesive joining, or when the study was outside the technical scope of low-temperature and room-temperature Au–Au bonding.

**Figure 3:**
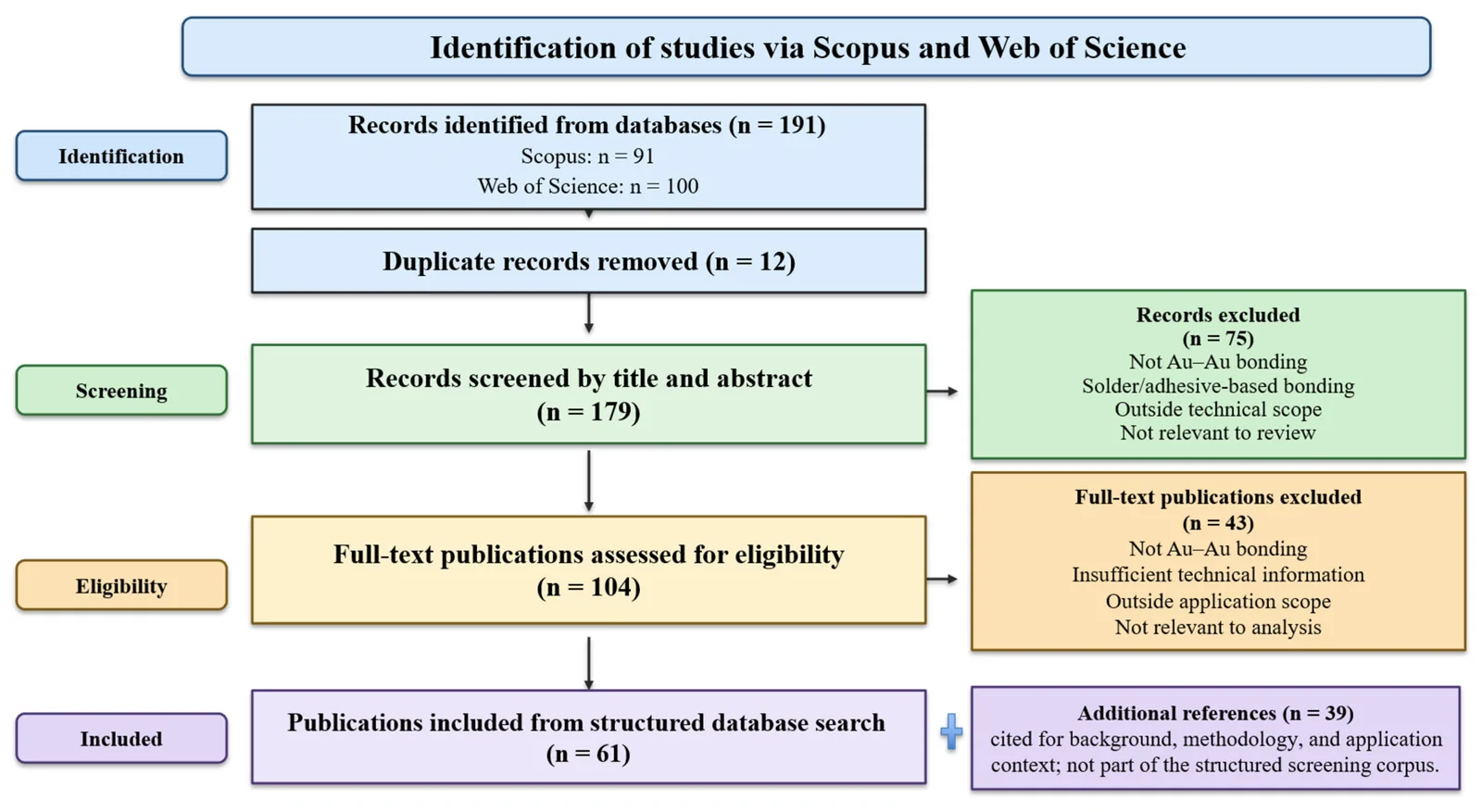
PRISMA flow diagram of the literature identification and screening process.

After title-and-abstract screening, 104 publications were assessed for eligibility. Of these, 43 were excluded after assessment because they did not satisfy the defined technical scope or did not provide sufficiently relevant evidence for the analysis. A total of 61 publications from the structured database search were retained for detailed review, see Figure 3. Additional references are cited throughout the manuscript to provide background on direct bonding, hybrid bonding, bonding mechanisms, metrology, and related integration technologies; these contextual references were not counted as part of the structured screening corpus.

This paper is organized as follows. Section 2 positions Au–Au bonding within advanced integration, including fine-pitch interconnects, wafer-level packaging, MEMS, flexible electronics, chiplet integration, and heterogeneous systems. Section 3 reviews the main solid-state Au–Au bonding routes, including thermocompression bonding, low-temperature direct bonding, surface roughness control, surface activation, contamination control, SAM-based protection, and intermediate-assisted Au-based bonding. Section 4 discusses device-level applications in optical and optoelectronic devices, MEMS hermetic and vacuum packaging, heterogeneous integration, RF/acoustic devices, and flexible hybrid electronics. Section 5 summarizes the metrology methods used to evaluate surface readiness, interface quality, mechanical strength, electrical functionality, and hermeticity. Section 6 analyzes the main reliability limits and performance constraints, including bonded-area yield, mechanical integrity, and electrical stability. Section 7 highlights research gaps and future directions for manufacturable Au–Au bonding in heterogeneous integration and flexible electronics.

## 2. Position of Au–Au Bonding in Advanced Integration

Au–Au direct bonding is important for advanced integration. It provides a direct metallic joining route for fine-pitch interconnects [13], wafer-level bonding, MEMS packaging, optical and optoelectronic microsystems [14], and heterogeneous integration. The main goal is to achieve high interconnect density. At the same time, Au–Au bonding avoids some limitations of solder-based bonding and oxide-forming metal interfaces. The following studies show how this role has expanded from interconnection toward broader wafer-level and heterogeneous integration applications.

The early literature positioned Au–Au bonding primarily as an enabling technology for high-density interconnects. An early work presented by [13], who introduced SAB for ultra-fine-density flip-chip and bumpless interconnect systems, confirms that the trend toward ultra-fine-density integration requires low-cost, ultra-high-density, low-damage, and low-temperature interconnect technology. They demonstrated direct Au–Au and Au–Cu bonding using their SAB approach. The same principle was later extended from interconnects to three-dimensional wafer-level packaging. Takegawa et al. [15] reported 3D wafer-level packaging of MEMS using surface-activated bonding and through-Si vias, confirming that SAB-based Au/Au bonding was achieved in a wafer-level packaging flow with TSVs, and that the resulting 3D interconnections showed good uniformity and high yield. This packaging and interconnect perspective was also extended in the literature to examine whether Au–Au bonding could support high-density interconnection on flexible substrates. Sinha et al. [16] describe direct bonding between gold-bumped flip-chip ICs and matching gold-plated copper pads, aiming to achieve very high I/O densities per unit area relevant to next-generation flexible electronic systems, and conclude that experiments suggested a metallurgical bond could form between the mating gold surfaces due to cold-welding. Sinha et al. [17] developed this point further by studying how bonding pressure, temperature, and time affect Au–Au bond strength, suggesting that bonding proceeds through plastic flattening of Au asperities followed by a time-dependent mechanism, with cold-welding presented as a possible explanation under the relatively low-temperature conditions examined.

More recent studies have examined this role further by considering chiplet integration and heat-transfer requirements. In these studies, Au–Au bonding is being suggested for heterogeneous integration and, in some cases, for thermal management. Sharma et al. [18] demonstrated solderless, passivation-free direct Au–Au pillar bonding by TCB, confirming that the developed interconnects can be used for the heterogeneous integration of CMOS and MEMS-based chiplets, and highlighting practical advantages of Au pillars, including the absence of intermetallic compound formation and the fact that Au does not oxidize like Cu. In addition, ref. [19] proposed Au–Au direct bonding for enhanced thermal management in heterogeneous integration, arguing for the use of gold as an inert bonding layer to reduce oxidation issues. Although these studies address different functions, both emphasize the value of an oxidation-resistant metallic interface in heterogeneous systems.

The literature also includes Au–Au bonding within the broader wafer-bonding theme and comparative studies of bonding metals. Vigna et al. [20] describe wafer-to-wafer bonding methods used in MEMS, considering thermocompression Au–Au among the permanent bonding approaches, including study cases for MEMS products. In addition, ref. [21] discuss Al–Al direct bonding and directly compare aluminum with gold and copper, declaring that Au–Au and Cu–Cu direct bonding are widely used and that oxide challenges are much smaller or more controllable in those systems than in Al. These comparisons strengthen the importance of surface chemistry when selecting a metal for direct bonding.

## 3. Bonding Routes for Solid-State Au–Au Joining

The reviewed solid-state Au–Au bonding routes show a clear transition. Early research directions relied mainly on thermocompression. Heat and pressure were used to create real contact between Au surfaces. Newer low-temperature and room-temperature routes depend more on surface preparation. These include surface smoothing, contamination control, and surface activation before bonding. Because TCB provides the historical and technical baseline for this transition, it is discussed as a technical baseline before the low-temperature and room-temperature routes that form the focus of this review.

### 3.1. TCB Baseline

Au–Au TCB is the most established route for solid-state Au–Au joining, where heat, pressure, time, and surface condition together determine the final bond quality. TCB is discussed for fine-pitch interconnects, wafer-level sealing, and device integration. Across the studies, the process is usually described through the effects of bonding temperature, bonding pressure, bonding time, and surface condition. The purpose of this subsection is not to provide an exhaustive review of TCB, but to identify the process principles and limitations that motivated the development of lower-temperature approaches.

Early studies mainly established the temperature and pressure conditions required for bond formation. Ang et al. [22] positioned Au–Au bonding as a direct metal bonding route for microsystems integration and reported that bonding occurs only above a threshold temperature, above which the tensile strength reaches a maximum with increasing bonding pressure. The follow-up paper by [23] examined temperatures from 100 to 300 °C and pressures from 200 to 600 g/bump, again reporting a critical bonding temperature below which bonding does not occur; above that temperature, shear strength increases because the true bonded area increases, and the paper interprets the critical temperature as the onset of breakup of organic barrier films. These studies show that conventional TCB depends on sufficient thermal and mechanical energy to remove interfacial barriers and increase the real bonded area.

Later studies therefore examined whether improved surface preparation could reduce the required bonding temperature. For instance, ref. [24] studied dodecanethiol self-assembled monolayers (SAMs) for Au–Au TCB at 80–180 °C and 225–566 MPa and concluded that the method achieved strong bonding at significantly lower temperature. Chin et al. [25] also reported bonded joint strength of 26.9 g per bump at 160 °C, with the role of SAMs described as passivating the Au surface before bonding. These results provide an early connection between TCB and the surface-engineering strategies discussed in the following subsections.

To explain macroscopic bonding results, several studies examined contamination removal, surface deformation, and void evolution at the interface. Unami et al. [26] studied vacuum-ultraviolet treatment before Au–Au flip-chip bonding and showed that this treatment removed carbon-based contaminants and improved average bump shear strength by a factor of 1.6. Goorsky et al. [27] investigated low-temperature, low-pressure Au–Au TCB at 150–250 °C and about 3 MPa, arguing that at 150 °C the void morphology was associated with the initial surface roughness; at higher temperatures the void length decreased and void height increased. The paper explains these changes in terms of increased surface Au diffusivity and reduced yield stress and elastic modulus at higher temperatures.

In parallel, TCB became an established sealing method for wafer-level and MEMS packaging. Park et al. [28] described TCB of electroplated gold for wafer-level hermetic packaging of RF-MEMS devices and reported complete wafer bonding at 320 °C and 2.5 MPa, with a leak rate of 2.74 ± 0.61 × 10^−11^ Pa·m^3^/s. Zoumpoulidis et al. [29] investigated die-to-wafer sealing by ultrasonic and thermocompression Au–Au bonding of narrow frames and found that thermocompression provided better bonding of the narrower features. Schjølberg-Henriksen et al. [30] measured residual gas pressure in sealed cavities and reported 0.18 mbar for cavities sealed by Au–Au TCB, with estimated maximum leakage rates between 10^−13^ and 10^−15^ mbar·l·s^−1^. In the same general direction, ref. [31] studied wafer-level Au–Au bonding at 350, 400, and 450 °C with pull-test bond strengths from 8 to 102 MPa, arguing that higher bonding temperature increased bond strength; eutectic reactions, grain growth, and TiW buckling were most pronounced at 450 °C, so bonding below the Au-Si eutectic temperature was recommended. These studies demonstrate the sealing capability of TCB, but they also show the high temperatures commonly required and the possibility of temperature-induced interfacial changes.

The established TCB process was also transferred to several devices and heterogeneous-integration platforms. Li et al. [32] used Au/Au direct wafer bonding at 260 °C for AlGaInP LEDs and reported a complete bonding structure without interface cracking. Karbownik et al. [33] investigated direct Au–Au die bonding for GaAs/AlGaAs quantum cascade lasers and reported good-quality direct Au–Au bonding, with parameters comparable to those of AuSn eutectic bonding. Sorensen et al. [34] evaluated direct Au–Au thermocompression bonding of InP dies onto a silicon interconnect fabric and reported shear strengths from 38 to 238 MPa as bonding pressure increased from 100 to 350 MPa, confirming that all attached dies withstood thermal cycling. Yang et al. [35] extended Au–Au TCB to superconducting interconnects and established bonding at 140 °C, with average shear force recorded above 100 N for 2 × 2 mm^2^ dies while retaining the superconducting transition temperature of Nb.

The more recent papers focused on the thermocompression theme in heterogeneous integration and advanced packaging. Wang et al. [36] proposed low-temperature wafer-level Au–Au thermocompression bonding at 230 °C for heterogeneous integration, using O_2_ plasma pretreatment and reporting good interface and electrical performance. Bargiel et al. [37] compared anodic bonding with Au–Au TCB for high-pressure micro cooling devices and found that thermocompression-sealed samples reached burst pressures up to 690 bar, compared with 530 bar for anodically bonded samples, although the thermocompression results were less homogeneous. Sharma et al. [18] reported solderless, passivation-free direct Au–Au pillar TCB for heterogeneous integration, recording an average shear strength of 128 MPa and electrical continuity across all daisy chains. Zhao et al. [38] used simultaneous Au–Au TCB and Si–Si direct bonding in a single heating process for wafer-level packaging of MEMS accelerometers, presenting a static capacitance yield above 77%, hermeticity yield above 90%, and average packaging strength of 26 MPa. Although these demonstrations confirm the significance of TCB, they also show that the acceptable thermal and pressure conditions depend strongly on the materials and devices being integrated.

Overall, TCB is a baseline for Au–Au joining. However, it usually depends on high temperature and pressure. This limitation motivates lower-temperature approaches based on surface engineering. The following subsections therefore shift the discussion from heat- and pressure-assisted contact formation toward surface roughness control, contamination management, and activation-assisted bonding.

### 3.2. Low-Temperature Surface-Engineered Direct Bonding

Low-temperature bonding reduces thermal stress. This is important when bonding dissimilar materials. It is also important for temperature-sensitive devices.

Unlike TCB, low-temperature bonding relies more strongly on surface preparation before contact. The following subsections examine how roughness control, surface activation, and contamination protection reduce the thermal and mechanical requirements of Au–Au bonding.

#### 3.2.1. Surface Roughness Control in Direct Bonding

Surface roughness is important because the two bonding surfaces must come into close contact over a sufficiently large area. Takagi et al. [39] showed theoretically that the surface roughness condition for successful bonding depends not only on the RMS roughness, but also on the lateral size of the surface features. Smaller-scale roughness is more difficult to accommodate because more elastic deformation is required for the surfaces to make contact. Their Si–Si experiments also showed that bonding quality decreased as the RMS roughness increased from about 0.3–0.6 nm to around 1 nm or higher. Although the numerical limits reported for Si should not be directly applied to Au–Au bonding, the study provides a useful general explanation for why both surface roughness and the measurement scale are important in room-temperature direct bonding.

For Au–Au bonding, the reported surface roughness ranges from below 0.5 nm for smooth sputtered films to tens or hundreds of nanometers for rough plated Au surfaces. Because rough surfaces reduce the real contact area, several studies have focused on preparing smoother Au surfaces before bonding. The reported approaches include surface flattening, smooth-film transfer, template stripping, and mechanical planarization.

Smooth sputtered Au films can bond at room temperature without additional smoothing steps. Electroplated Au, however, is usually much rougher. This is important because many practical seal rings, bumps, and interconnects are made by plating. For electroplated Au, deliberate roughness reduction is therefore a prerequisite for room-temperature bonding. Goto et al. [40] further extended roughness and geometry control to low-pressure bonding using hollow pyramidal and flat-topped Au micro-bump arrays.

An early example is [41], in which they reported room-temperature direct bonding of electroplated Au patterns flattened by a thermal imprint process. Their paper argues that an ultra-flat sapphire wafer was pressed onto the rough electroplated Au surface for 10 min at 200 °C under 150 MPa. The flattened Au patterns were bonded by surface-activated room-temperature bonding and bonding strength above 200 MPa was obtained. The paper also confirms that narrow multi-wall line patterns were designed to ensure bonding strength and hermeticity. The bonding strength was further improved by combining flattening with the multi-wall narrow pattern.

Subsequent studies shifted from directly flattening the plated Au to transferring a smooth Au surface onto it. A lift-off replication process was used by [42] to replicate an atomically smooth surface onto electroplated Au patterns. They obtained an RMS roughness of 0.8 nm, enabling room-temperature Au–Au bonding in atmosphere with bonding strength of about 250 MPa. Kurashima et al. [43,44] then evaluated room-temperature bonding of electroplated Au seal rings with surfaces smoothed by lift-off and imprint methods. They showed that smoothed surfaces produced strong bonding while rough electroplated surfaces produced very weak bonding. The paper reports good hermetic sealing when the smooth surface was replicated by the lift-off method. Kurashima et al. [45] further demonstrated the direct transfer of an atomically smooth Au thin film onto electroplated Au patterns, obtaining a transferred surface with RMS roughness of 0.6 nm and bonding strength of about 225 MPa at room temperature in atmosphere. Kurashima et al. [46] continued this strategy by directly transferring an atomically smooth Au film onto sealing-ring patterns and then bonding them at room temperature in atmospheric air. The bonding strength was about 225 MPa, almost the same as TCB at 200 °C. These studies show that a smooth Au bonding surface can be formed on top of a rough electroplated structure.

Building on this approach, later work examined how the thickness of the transferred Au film affects the final surface roughness. The role of transferred smooth Au is clarified by [47]. The study demonstrates that Au films deposited on smooth SiO_2_ with RMS roughness of 0.24 nm were investigated for direct transfer to rough Au surfaces. When continuous Au films with thicknesses of 20, 51, or 102 nm were transferred, the RMS roughness of the rough Au surfaces decreased from 1.6 nm to 0.4 nm. In contrast, transferred Au films thinner than 5 nm increased the surface roughness. More discussion is provided by [48], in which they reported a multiple thin-film transfer process based on template stripping. Their paper gives direct quantitative evidence that repeated transfer reduced roughness from 205 nm to 10 nm over a 90 × 90 μm^2^ area and from 3.1 nm to 0.8 nm over a 1 × 1 μm^2^ area. The paper also shows that Au–Au surface-activated bonding at 150 °C produced strong bonds only when the template-stripped Au films were used.

**Figure 4:**
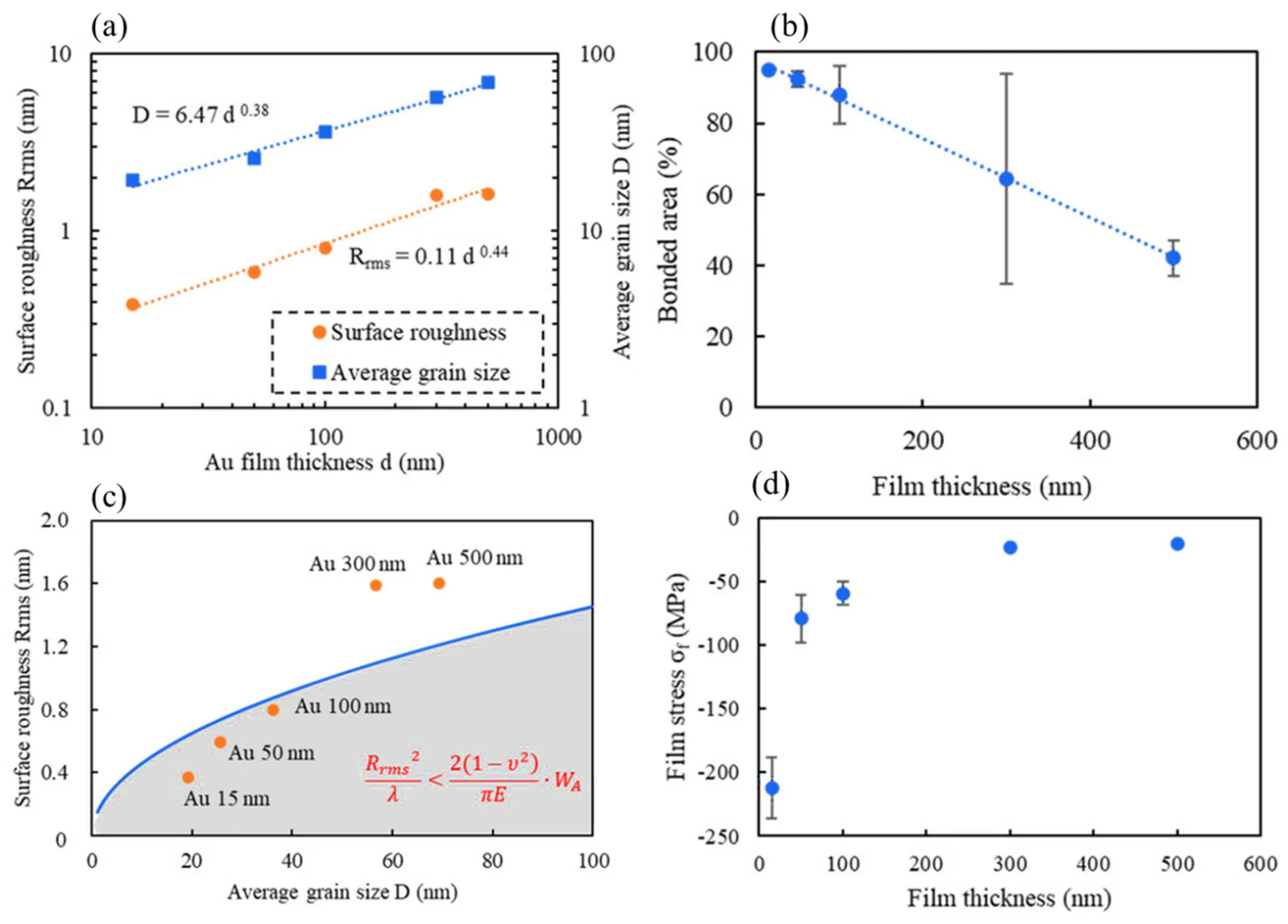
Thickness of the film and surface roughness impact, (**a**) surface roughness and average grain size, (**b**) bonded area, (**c**) relationship between surface roughness and average grain size, and (**d**) film stress [49].

The same roughness and bonding relationship was then evaluated at the wafer scale. This wafer-scale perspective is given by [49], as shown in Figure 4, who examined the effects of Au film thickness and surface roughness on room-temperature wafer bonding and wafer-scale vacuum sealing by Au–Au SAB. According to this study, for Au film thicknesses of 15–500 nm and surface roughness of 0.3–1.6 nm, the bonded area exceeded 85% when the Au thickness was 100 nm or less. Wafer-scale vacuum sealing was achieved when the Au thickness was 50 nm or less.

**Figure 5:**
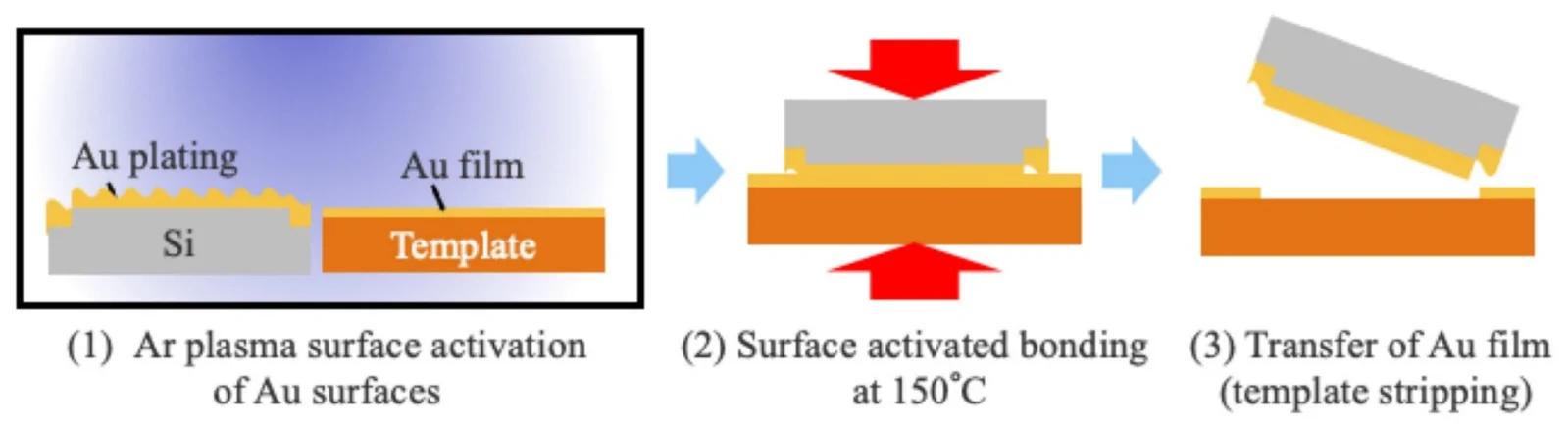
Smoothing with polyimide template [52].

Recent studies have adapted smooth-film transfer to rough plated Au using polymer templates. Koseki et al. [50] reported that transferring Au thin films from polyimide and SiO_2_/Si templates reduced the RMS roughness of rough plated Au surfaces from 21 nm to sub-nanometer. Koseki et al. [51] then reported that, for rough plated Au bumps, the RMS value was reduced from 30 nm to 6 nm by transferring Au thin films from a polyimide template. Takeuchi et al. [52] reported a smoothing strategy based on polyimide template stripping combined with surface-activated bonding for room-temperature direct Au bonding; see Figure 5. Transferring Au films from a polyimide template three times reduced the RMS roughness of plated Au from 21 nm to 5 nm, and produced fewer interfacial gaps than a SiO_2_-template route. The bonding strength was described as high as bulk fracture.

**Figure 6:**
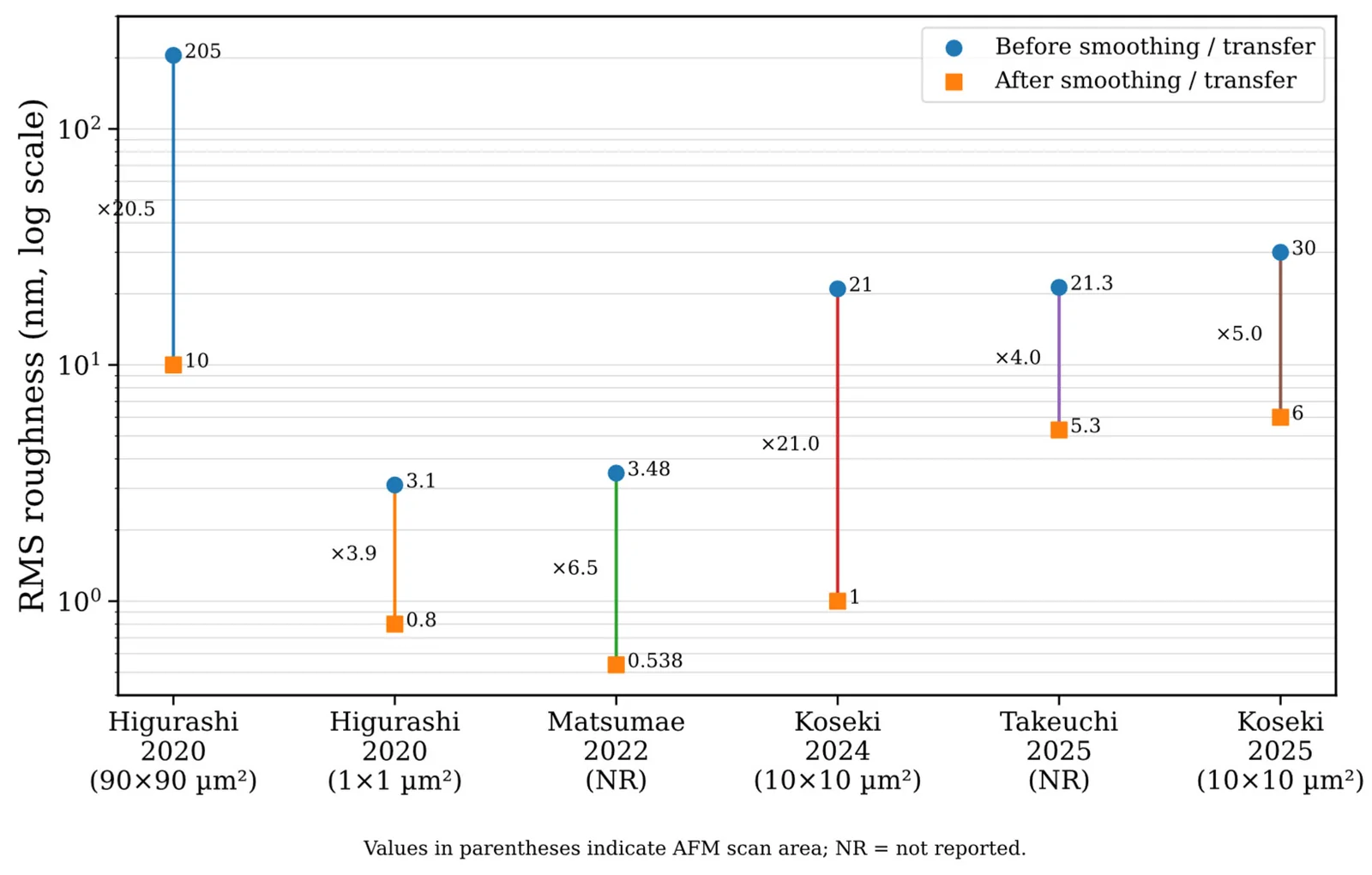
Roughness reduction pathways enabling low-temperature Au–Au bonding, plotted according to data from [48,50,51,52,53].

Figure 6 shows how smoothing and transfer methods reduce Au surface roughness. The reduction is large for rough plated Au surfaces. In several studies, the roughness decreased from tens or hundreds of nanometers to a few nanometers or below 1 nm. This explains why surface smoothing is important for low-temperature Au–Au bonding. A smoother Au surface increases real contact area and makes bonding easier at lower temperature.

In addition to thin-film transfer, mechanical planarization has been used to prepare plated Au structures for bonding. Hirano et al. [54] used planarized electroplated Au bumps for wafer-level vacuum sealing and interconnection. They recommended a wafer-level Au–Au bonding flow that begins with separate preparation of a non-planar wafer and a planar wafer. For the non-planar wafer, an Au/Ti seed layer is first deposited, followed by Au electroplating, seed-layer removal, vacuum annealing above 300 °C, planarization by fly cutting, and resist removal. For the planar wafer, the process consists of Au/Ti metal deposition followed by metal patterning. After both wafers are prepared, bonding and sealing are carried out by Ar-plasma surface activation, wafer alignment, and final bonding in vacuum at temperatures above 250 °C.

In general, these studies show that roughness reduction is a primary enabling step for low-temperature and room-temperature bonding. However, geometric smoothness alone is not sufficient; the prepared Au surface must also be chemically clean and active at the moment of contact.

#### 3.2.2. Surface Activation Methods for Low-Temperature Au–Au Bonding

Surface activation enables low-temperature and room-temperature Au–Au bonding. It removes adsorbed contaminants from the Au surface and can also modify the surface chemistry, so that the Au surface becomes more reactive before contact. The reviewed plasma activation methods differ in terms of gas chemistry and the pressure regime under which the plasma is generated. Low-pressure and atmospheric-pressure plasmas interact with Au surfaces through different mechanisms. Under reduced pressure, energetic Ar species can contribute to physical sputtering and removal of surface contaminants, whereas at atmospheric pressure, frequent gas-phase collisions substantially reduce the contribution of energetic physical bombardment. The following discussion therefore distinguishes plasma treatments first according to their operating pressure and then according to their gas chemistry.

- Low-Pressure Plasma and Beam Activation

Low-pressure Ar plasma and fast-atom-beam activation are established approaches for Au–Au surface-activated bonding. Under reduced-pressure conditions, energetic Ar species can remove adsorbed contaminants through physical bombardment and sputtering. Because Au does not form a stable native oxide under ordinary conditions, this treatment can expose a clean Au surface without introducing a persistent oxide layer. This physical activation mechanism distinguishes conventional low-pressure Ar-based SAB from atmospheric-pressure plasma treatments.

B. Ar Plasma and Fast-Atom-Beam Activation

An early paper is [13], in which the authors introduced SAB for new flip-chip and bumpless interconnect systems. Their paper claims that the method was developed for low-cost, ultra-high-density, low-damage, and low-temperature interconnect technology. In that process, the bonding pads and bumps are cleaned by Argon (Ar) plasma or atom beam irradiation, aligned precisely, and then bonded directly by contact at low temperature. The paper also declares that preliminary experiments demonstrated Au–Au and Au–Cu direct bonding. Higurashi et al. [55] reported low-temperature flip-chip bonding of a vertical cavity surface emitting laser (VCSEL) on a micromachined Si substrate. Their paper confirmed that the Au electrodes were cleaned by Ar radio frequency (RF) plasma. The Au–Au bonding was achieved by contact in ambient air, and at 100 °C the die-shear strength exceeded the MIL-STD-883 failure criterion. In another work, ref. [56,57] reported low-temperature bonding of LiNbO_3_ waveguide chips to Si substrates by introducing surface activation into the flip-chip process. Their work shows that the Au surfaces were cleaned by Ar RF plasma. Au–Au bonding was carried out in ambient air with applied static pressure. Successful bonding was achieved at relatively low temperatures, while higher temperatures caused cracking because of coefficient-of-thermal-expansion (CTE) mismatch. Imamura et al. [58] reported SAB of flip-chip VCSELs to Si substrates and achieved a die-shear strength of 84 MPa at 150 °C, and Imamura et al. [59] further showed low-temperature direct bonding of flip-chip mountable VCSELs, reporting die-shear strength above 50 gf (54 MPa) at 150 °C and normal L-I-V characteristics after bonding.

Early optical-microsystem studies showed that Ar RF plasma activation can clean Au electrodes and enable low-temperature Au–Au bonding in ambient air. Higurashi et al. [55], Takigawa et al. [56,57], and Imamura et al. [58,59] used this route to demonstrate that plasma activation can lower the bonding temperature by improving surface cleanliness before contact. Takigawa et al. [60] further used Ar RF plasma activation before low-temperature Au–Au bonding in ambient air, showing the continued use of plasma-assisted bonding for optical microsystem assembly.

The literature then extends toward room-temperature Au–Au bonding in ambient air. Takigawa et al. [61] demonstrated room-temperature bonding of VCSEL chips on Si substrates using Au microbumps after Ar RF plasma activation. The paper reports no significant degradation in L-I-V characteristics after bonding.

A second major development in the literature is the use of ultrathin Au films for wafer-scale room-temperature bonding. Kunimune et al. [62] introduced room-temperature wafer bonding for MEMS packaging. The study used patterned Au thin films with RMS roughness below 0.5 nm after Ar RF plasma activation. Their experiment concludes that, with plasma treatment, high bonding energy was obtained regardless of exposure time to air or ethanol after Au deposition. Higurashi et al. [63] further reported room-temperature bonding of wafers with smooth Au thin films in ambient air using SAB. According to this study, wafers with Au thin films having RMS roughness below 0.5 nm and thickness below 50 nm were successfully bonded at room temperature. The bonded wafers showed die-shear strength of 47–70 MPa without heat treatment, and transmission electron microscopy showed direct bonding on the atomic scale. Okumura et al. [64] studied the influence of air exposure time on bonding strength in Au–Au surface-activated wafer bonding. They show that synthetic quartz wafers with smooth Au thin films of 30 nm thickness and 0.43 nm RMS roughness were successfully bonded in air at room temperature after Ar RF plasma activation, even after long air exposure times of 800–2000 h following Au deposition. A high die-shear strength was obtained, and fracture typically occurred inside the bulk glass rather than at the bonded interface.

Yamamoto et al. [65] demonstrated room-temperature wafer bonding of 4-inch Si wafers with thin Au films for integrated plasmonic devices using Ar plasma surface activation. The study emphasizes that low-temperature bonding is desirable because higher-temperature annealing caused Cr diffusion in the multilayer metal films. The literature also includes several studies that compare or optimize the plasma activation conditions used in SAB.

These studies establish reduced-pressure Ar-based activation as an important physical surface-cleaning route for Au–Au SAB, particularly when combined with sufficiently smooth Au surfaces. Atmospheric-pressure plasma treatments, however, operate under different discharge conditions and are therefore discussed separately below.

C. Oxygen-Containing Plasma and Oxidation

O_2_ plasma treatments show that not all surface activation improves Au–Au bonding. The chemical oxidation of Au can form Au_2_O_3_ and weaken the interface unless the oxide is removed by subsequent annealing or treatment.

Yamamoto et al. [66] analyzed Ar- and O_2_-plasma-treated Au surfaces for room-temperature wafer-scale Au–Au bonding. Their paper demonstrates that O_2_ plasma formed Au_2_O_3_ on the surface and led to weak bonding, while Ar plasma produced strong bonding and caused fracture in the bulk wafers during blade testing. This comparison was developed further by [67], who investigated wafer-scale Au–Au bonding using ultrathin Au films in ambient air. They confirmed that Ar plasma mainly activates the surface through physical etching, while O_2_ plasma acts mainly through chemical oxidation. In this case, Ar-plasma-treated samples showed much stronger bonding, while O_2_-plasma-treated samples reached only about 0.1 J/m^2^ bond strength. Ogino et al. [68] compared Ar plasma, O_2_ plasma, and UV/O_3_ treatment and concluded that high bonding strength was obtained only with Ar plasma. Earlier pretreatment studies also support the importance of surface cleaning before low-temperature Au–Au bonding.

O_2_-containing plasma often weakens Au–Au bonding because it can form a surface oxide. This limitation led researchers to explore non-oxidizing atmospheric plasmas that clean and activate Au without leaving a persistent oxide layer.

D. Atmospheric-Pressure Plasma Activation

Atmospheric-pressure plasma provides a distinct route for surface preparation before low-temperature Au–Au bonding. Compared with low-pressure plasma, the higher collision frequency at atmospheric pressure reduces the contribution of energetic physical sputtering. Consequently, gas chemistry and plasma-generated reactive species become particularly important. Atmospheric-pressure treatments are therefore considered separately from the low-pressure Ar activation discussed above.

E. Ar and Ar/H_2_ Atmospheric-Pressure Plasma

Atmospheric-pressure Ar/H_2_ plasma has been investigated for room-temperature and low-temperature Au–Au bonding because it can remove surface contamination and modify the Au surface before bonding.

Higurashi et al. [69] reported low-temperature Au–Au bonding using Ar/H_2_ atmospheric-pressure plasma treatment for optical microsystems. This study applies room-temperature bonding of semiconductor chips with Au thin films to coined Au stud bumps with smooth surfaces (Ra: 1.3 nm). The die-shear strength exceeded the requirement of MIL-STD-883F method 2019, and device characteristics showed no degradation. Another relevant work [70], also demonstrated room-temperature bonding of optoelectronic chips with Au thin film to coined Au stud bumps using an Ar and hydrogen gas mixture atmospheric-pressure plasma, with no degradation in optical or electrical device characteristics. In [71], the authors demonstrated wafer-scale Au–Au SAB using atmospheric-pressure plasma, with the entire process performed in ambient air. Their paper confirms that only partial bonding was obtained after 2.5 s plasma treatment, while strong bonding was obtained after 10 s and 30 s treatments; Si substrates were sometimes broken in the razor blade test.

These studies should therefore be considered separately from low-pressure Ar physical activation, even when Ar is used in both processes, because the pressure regime substantially changes the dominant surface-interaction mechanism.

F. N_2_ Atmospheric-Pressure Plasma

N_2_ plasma provides a non-oxidizing atmospheric-pressure activation route for Au–Au bonding. This plasma-chemistry variant was reported by [72], who investigated nitrogen atmospheric-pressure plasma treatment for low-temperature Au–Au bonding in optical microsystems. In the Ar+O_2_ case, oxidation of the Au surface was detected as Au_2_O_3_, whereas no oxidation was observed after N_2_ plasma treatment. The paper also shows that N_2_ atmospheric-pressure plasma improved Au–Au bonding compared with Ar+O_2_ plasma at a temperature of 150 °C.

These results show that, within the atmospheric-pressure regime, gas chemistry strongly affects the Au surface condition and subsequent bonding behavior.

G. VUV, UV/O_3_, and Sequential Photochemical–Plasma Activation

VUV- and UV/O_3_-based treatments introduce a photochemical activation pathway, but the strongest results are generally obtained when photochemical cleaning is combined with Ar plasma or mild heating. Okada et al. [73] reported low-temperature Au–Au flip-chip bonding with VUV/O_3_ treatment for 3D integration and achieved bonding at 200 °C. Ogino et al. [74] demonstrated pressureless wafer bonding with low-roughness Au thin films in air, using sequential VUV-light followed by Ar-plasma treatment. Ogino et al. [75] reported that VUV irradiation combined with mild heating below 150 °C for 5 min enabled surface-activated bonding of Au thin films.

These studies suggest that photochemical treatments are the most effective when used as a part of a sequential activation strategy rather than as a standalone replacement for physical plasma activation.

H. WVPAB for Rough and Flexible Substrates

WVPAB expands Au–Au direct bonding to rougher electrodes and flexible polymer substrates, where conventional SAB requirements are difficult to satisfy. It extends the accessible surface condition range to rough electrodes and flexible polymer substrates, partially relaxing the roughness constraint that limits conventional SAB. Takakuwa et al. [7] developed WVPAB for direct Au bonding on ultrathin polymer films in flexible electronics. Water-vapor plasma at 50 W for 40 s was applied to Cr/Au electrodes on parylene substrates with RMS roughness of 6.29 nm, far above the sub-nanometer threshold required for conventional SAB. Bonding was achieved at room temperature with only 2 N applied for 5 s, and peel testing resulted in substrate failure rather than interfacial separation. Electrical resistance changed by less than 1% after 10,000 bending cycles. Takamatsu et al. [76] developed WVPAB for direct Au bonding of gold electrodes on ultrathin polymer films, specifically targeting flexible integrated electronics. A device-level application is introduced in [76].

WVPAB is therefore especially important for FHE because it relaxes the surface roughness constraint while preserving direct metallic bonding and mechanical flexibility.

Across the reviewed methods, the activation literature shows that non-oxidizing treatments, especially Ar-based activation, are the most consistently effective, while oxidizing treatments require careful control to avoid weakening the Au–Au interface. The effectiveness of activation also depends on how well the cleaned surface is protected before bonding.

#### 3.2.3. Contamination Control and SAM-Based Surface Protection

Contamination control is essential for Au–Au bonding because adsorbed organic and oxygen-containing species can block direct metallic contact even when the Au surface is geometrically smooth.

The use of SAMs as a chemical aid for Au–Au bonding is applied mainly in two ways. The first is to support low-temperature TCB by limiting surface contamination before bonding. The second is to protect activated Au surfaces so that room-temperature bonding remains possible after a delay between activation and contact. For the second use, ref. [77] used a SAM to protect an activated Au surface intended for room-temperature bonding. The paper shows a specific limitation of surface-activated bonding: the effect of activation is short-lived, because the fresh Au surface can lose bonding ability after exposure to air. The SAM-based protection strategy was proposed to extend that usable window and preserve the bonding capability of the activated Au surface until joining. Thus, SAM-supported strategies show that chemical surface protection can complement mechanical smoothing and plasma activation, especially when there is a delay between surface preparation and bonding.

#### 3.2.4. Intermediate-Assisted Low-Temperature Au-Based Bonding

Not all approaches that reduce bonding temperature preserve a purely direct Au–Au interface. A separate group of studies introduces an intermediate layer to assist bond formation. Intermediate-assisted Au-based bonding has also been explored as a route to reduce bonding temperature and pressure. Fang et al. [78] proposed an Au–Au bonding route using Ag nanoparticles as a surface-modification layer. This enables bonding at 200 °C for 3 min under 30 MPa, reporting average bonding strength above 10 MPa together with void-free interface observations by TEM. In a follow-up study, ref. [79] applied the same idea to die attachment in power device packaging, reporting low-temperature Au–Ag NP–Au bonding at 200 °C for 3 min under 20 MPa without annealing.

Intermediate-assisted Au-based bonding broadens the low-temperature bonding landscape, but it should be distinguished from direct Au–Au bonding because the final interface includes an added nanoparticle modification layer. It is therefore included here as a related low-temperature strategy rather than as a direct Au–Au bonding route.

**Figure 7:**
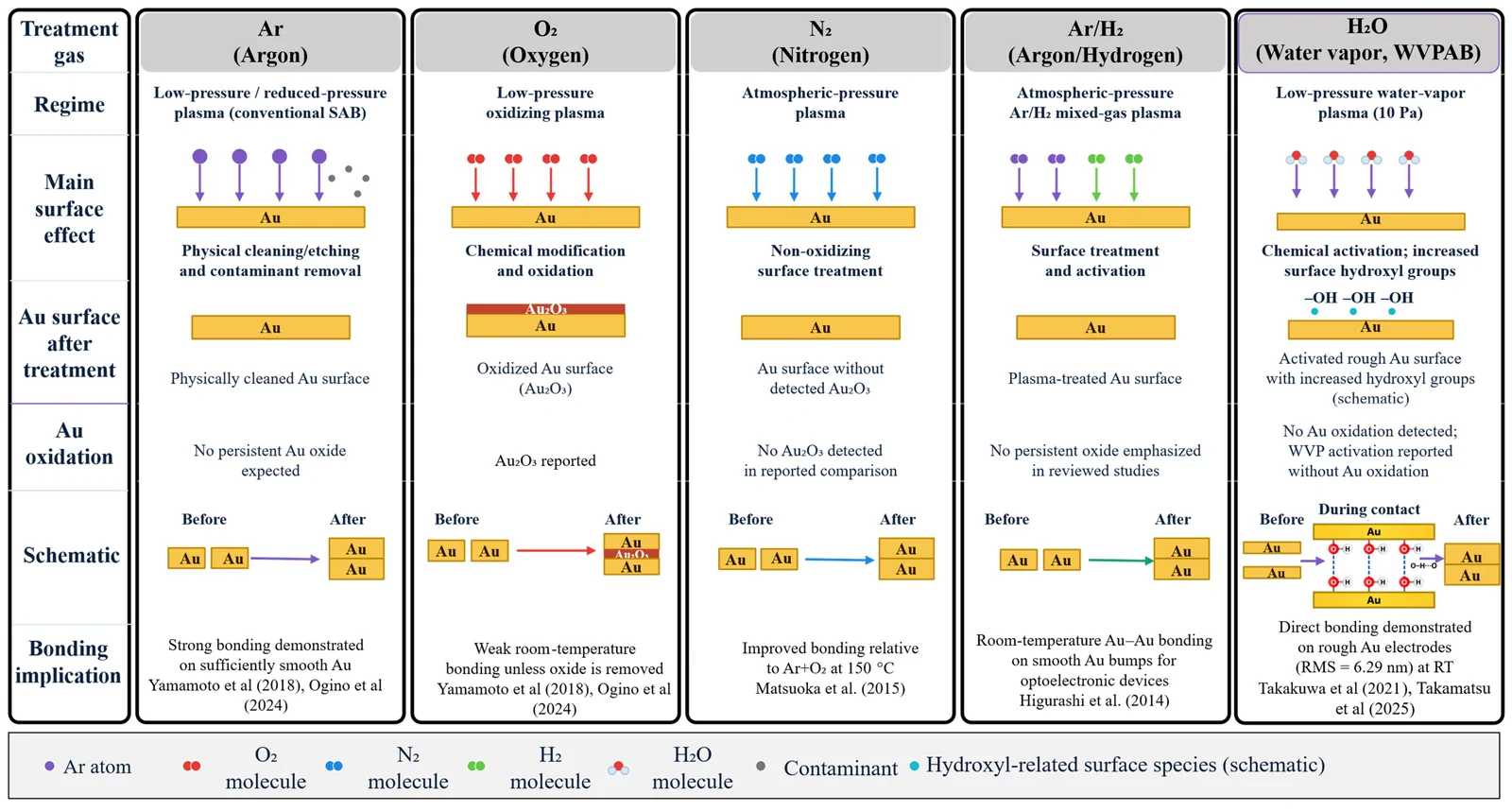
Comparison of activation gases (Ar, O_2_, N_2_, Ar/H_2_) and WVPAB treatment for Au–Au bonding, showing reported surface effects, oxidation state, and bonding outcomes [7,66,67,68,69,70,71,72,76,77].

Among studies using comparable experimental platforms, Ar activation produced substantially stronger bonding than O_2_-based treatment [66,67,68], consistent with physical cleaning by Ar and oxidation during O_2_ treatment. N_2_ atmospheric-pressure plasma avoided the Au oxidation observed for Ar+O_2_ treatment and improved Au–Au bonding at 150 °C [72]. Ar/H_2_ atmospheric-pressure plasma also enabled successful low-temperature and room-temperature bonding [69,70,71]. Together, these studies indicate that non-oxidizing activation routes are generally more favorable for maintaining a bondable Au surface, while the effectiveness of each treatment also depends on the plasma regime and initial surface condition. Figure 7 summarizes these surface activation treatments and their reported effects.

### 3.3. Mechanisms of Au–Au Bonding

Au–Au bonding involves several related processes. First, contaminants and other surface barriers must be removed or disrupted so that the Au surfaces can approach each other. In SAB, plasma or beam activation is mainly used for this purpose [13,59,66,67]. In TCB, heating can help remove or disrupt interfacial contamination, while pressure deforms surface asperities and increases the real contact area [23]. Second, sufficient real contact area must be created. Applied pressure and elevated temperature promote deformation of Au asperities, increasing the fraction of the nominal area that is in contact [17,23,27]. Room-temperature SAB cannot rely on thermally assisted deformation to the same extent, which explains the stronger requirement for initially smooth surfaces [39,41,63].

Once clean Au surfaces make contact, metallic interactions can develop across the interface [63,80]. Several low-temperature studies describe this behavior in terms of cold welding, particularly where Au asperities deform and clean metallic regions contact without melting [16,17,80]. Cold welding should, however, be regarded as an interpretation of intimate metallic contact rather than a mechanism demonstrated identically in every Au–Au bonding configuration. With increasing temperature and bonding time, surface and interfacial atomic diffusion become increasingly important [27,31].

## 4. Device-Level Applications of Au–Au Low-Temperature Bonding

Au–Au direct bonding has been applied in device systems where low thermal budget, thin metallic interfaces, and stable electrical or mechanical performance are required. The reviewed application studies can be grouped into optical and optoelectronic devices, MEMS hermetic and vacuum packaging, heterogeneous integration, and FHE. These application groups illustrate how the same bonding principles are adapted to different thermal, mechanical, and functional requirements. 

### 4.1. Optical and Optoelectronic Devices

Optical and optoelectronic devices are a major application area for low-temperature Au–Au bonding because they often combine materials that are sensitive to CTE mismatch. For LiNbO_3_/Si waveguide integration, low-temperature Au–Au bonding helped reduce cracking problems and later supported passive alignment with low optical loss [56,57,60]. For VCSELs and related optoelectronic chips, the bonded devices retained normal L-I-V, optical, or electrical characteristics after bonding [13,55,58,59,61].

Beyond waveguides and VCSELs, the same low-temperature joining principle has been applied to lasers, silicon photonics, and display devices. Rantamaki et al. [14] reported a low-temperature gold-to-gold bonded semiconductor disk laser assembled with a diamond heat spreader. A high-power optoelectronic application was reported by [81], who demonstrated low-temperature GaAs/SiC wafer bonding with a Au thin film for high-power semiconductor laser applications. A more recent study [82] extended this approach to room-temperature GaAs–SiC wafer bonding using a thin Au film. Usui et al. [83] proposed solder-free low-temperature flip-chip integration for silicon photonic platforms using gold-stud bump bonding. A further application example was reported by [84], who fabricated bottom-emitting OLED panels interconnected with an encapsulation substrate by Au–Au flip-chip bonding followed by capillary-driven filling.

These studies show that Au–Au bonding is especially useful for optical and optoelectronic integration when device performance must be preserved under a low thermal budget.

### 4.2. MEMS Hermetic and Vacuum Packaging

The role of Au–Au bonding extends beyond device attachment to packages that must also provide sealing and vacuum stability. MEMS hermetic and vacuum packaging is another important application area for low-temperature and room-temperature Au–Au bonding because the bonding interface can provide mechanical attachment, electrical interconnection, and sealing within the same package architecture. Takegawa et al. [15] showed that SAB-based Au/Au bonding can be integrated into 3D wafer-level MEMS packaging with through-Si vias. Other studies showed that smooth Au thin films and smoothed electroplated Au seal rings can support room-temperature wafer bonding and hermetic sealing for MEMS packages. Studies using smooth Au films and smoothed electroplated Au seal rings [43,46,49,62] further demonstrated room-temperature bonding and, in selected configurations, hermetic or vacuum sealing.

These surface-engineering approaches were subsequently evaluated in larger-scale packaging and functional microsystem demonstrations. In a wafer-level packaging study, ref. [85] reported hermetic packaging of MEMS resonator components using interposer wafers with TSVs and cap wafers, where more than 5000 quartz resonator components were assembled onto each interposer wafer by Au–Au direct metal bonding; of 4824 tested devices, more than 75% were properly sealed under vacuum. Matsumae et al. [86,87] showed that barrier-layer selection is important for MEMS packages that require vacuum degas annealing before Au–Au SAB. Matsumae et al. [53] considered room-temperature Au–Au bonding toward vacuum-packaged microsystems by combining direct bonding, hermetic sealing, and getter functionality. Yildiz et al. [88] used Au–Au bonding of LTCC vias and bumps during anodic bonding in capacitive micromachined ultrasonic transducer (CMUT) fabrication and packaging, showing that Au-based joining can be integrated into functional microsystem fabrication flows.

The abovementioned studies show that Au–Au bonding can serve as a multifunctional MEMS packaging interface, combining wafer-level sealing, vacuum compatibility, interconnection, and mechanical integrity.

### 4.3. Heterogeneous Integration and RF/Acoustic Devices

Beside hermetic packaging, low-temperature Au–Au bonding is also used for heterogeneous integration, especially when dissimilar materials must be joined without excessive thermal load. Ballandras et al. [89] used Au/Au direct bonding to fabricate LiTaO_3_/Si composite wafers for RF resonators and reported a strong reduction in thermal drift.

This material-integration advantage is particularly relevant to RF and acoustic systems that combine piezoelectric materials with silicon electronics. Another device-integration direction involves combining surface acoustic wave devices with large-scale integrated circuits and CMOS electronics. Park et al. [90] reported a wafer-bonding-based heterointegration process in which a lithium niobate SAW resonator was bonded to a BiCMOS wafer by Au–Au bonding after Ar-plasma activation, with the process designed to avoid CTE mismatch between LN and Si. Park et al. [90,91] and Tanaka et al. [92] then reported direct integration of a LiNbO_3_-based SAW resonator with a CMOS sustaining amplifier, again using low-temperature Au–Au bonding following plasma surface activation, achieving a 500 MHz one-chip SAW oscillator.

The literature then expanded from wafer-level material integration to selective die transfer, 3D assembly, and thermal-interface applications. Hikichi et al. [93] reported a wafer-level selective transfer method for FBAR-LSI integration, in which 1 mm × 1 mm FBAR dies were selectively transferred onto 2 mm × 2 mm BiCMOS sustaining amplifiers by low-temperature Au–Au bonding. Okada et al. [73] showed its use for 3D flip-chip integration, while [16,17] discussed Au-bumped flip-chip interconnects as a route toward high-I/O-density electronic systems. Shou et al. [19] further extended the application direction toward thermal management in heterogeneous integration.

This group of studies shows that Au–Au bonding can enable compact heterogeneous device stacks where electrical connection, mechanical joining, thermal stability, and material compatibility must be achieved together.

### 4.4. Flexible Hybrid Electronics

The need for a low thermal budget and thin interconnect becomes even more critical when the bonded structure must remain flexible. FHE requires thin bonding interfaces that remain stable during bending and repeated deformation. Water-vapor plasma-assisted Au–Au bonding has been used to join Au electrodes on ultrathin polymer substrates and to connect ultrathin silicon sensors with parylene wiring, showing its value for flexible integrated systems [7,76]. Low-temperature Au direct bonding has also been extended to Au-coated nanomeshes for flexible and breathable electronics [94]. These studies show that Au–Au bonding can support mechanically compliant, electrically stable, and thin interconnects for FHE.

## 5. Metrology for Au–Au Bonding

The literature shows that metrology for Au–Au bonding is usually organized around three questions. Is the Au surface ready to bond? Did a continuous interface actually form? Does the bonded structure still perform its intended function? In the reviewed papers, the first question is addressed mainly through surface roughness and surface condition. The second is addressed through cross-sectional interface analysis and bonded-area evaluation. The third is addressed through mechanical strength, electrical functionality, or hermetic sealing, depending on the application. These three questions provide the framework for comparing the metrology approaches used across the reviewed studies.

The evaluation generally begins before bonding by examining surface readiness. The most frequently reported metrology category is surface topography. In many low-temperature studies, roughness is not just a descriptive property; it is treated as a process-limiting variable. The studies use roughness data to show whether a rough electroplated or deposited Au surface has been converted into a bondable surface. In this part of the literature, roughness measurement is often the first screening tool because the success of later room-temperature or low-temperature bonding depends strongly on surface morphology.

Surface-roughness values reported in the literature require careful comparison because different roughness parameters and measurement conditions are used. The arithmetic average roughness, $R_{a}$ is the mean of the absolute height deviations from the mean surface, whereas the root-mean-square roughness, commonly reported as RMS or $R_{q}$ is the square root of the mean squared height deviations. Because larger deviations are weighted more strongly in the RMS calculation, the two parameters should therefore not be treated as numerically equivalent. More importantly, roughness values obtained using different AFM scan sizes, spatial resolutions, filtering procedures, or measurement instruments may represent different spatial scales of the surface. Consequently, the roughness values compiled in this review are reported using the parameter and measurement area used in the original study and are not converted directly between $R_{a}$ and RMS.

After surface readiness is established, the next step is to determine whether bonding produces a continuous interface. Here, cross-sectional imaging is used to determine whether the interface is continuous, whether voids or visible seams remain, and in some cases whether grain continuity develops across the bonded region. TEM is introduced in studies such as [63], while TEM/FIB is used in [76] for checking interface continuity after WVPAB.

Interface continuity alone, however, does not confirm that the bonded structure satisfies its intended function. The functional validation depends on the target application. For packaging and sealed structures, the key measurements are residual gas pressure, leak performance, or sealing yield, as introduced in [30,49,85]. For interconnects or devices, the outcome is often shown by die-shear or peel strength, electrical continuity, or preservation of device behavior after bonding.

Because individual studies report different combinations of surface, interface, and functional measurements, a quantitative comparison is needed. Table A1 (Appendix A) summarizes the main quantitative values reported in representative Au–Au bonding papers. The table focuses on process conditions, surface or geometry metrics, bond-strength indicators, and electrical, hermetic, or reliability outcomes. This format highlights the wide variation in reported evaluation methods while also showing the numerical ranges associated with successful Au–Au bonding, including roughness levels, bonding temperature, pressure or load, bonding time, bonded-area metrics, strength values, leak rates, and reliability outcomes.

The reviewed evidence indicates that successful Au–Au bonding cannot be judged by a single measurement, but requires linking surface preparation, interface structure, bond strength, and device-level performance.

## 6. Reliability Limits and Performance Constraints

The reliability of Au–Au bonding depends on three connected outcomes: achieving a sufficiently bonded area, maintaining mechanical integrity, and preserving electrical performance during operation.

### 6.1. Yield Limiters

A packaging-oriented reliability perspective is presented in [95]. They noted that direct Au surface treatment can reduce electrical resistance and signal loss, but practical implementation remains limited by reliability and process-integration challenges. This broader packaging perspective reinforces that Au–Au bonding must be evaluated beyond initial bond formation. At the bond-formation level, the literature identifies surface roughness, film geometry, activation state, and alignment as the main yield limiters.

Across the reviewed studies, bonded-area yield is controlled first by whether sufficient real metal-to-metal contact can be created at the moment of joining. Surface roughness is the most consistent limitation because local asperities prevent uniform contact and leave unbonded regions. The smoothing and transfer studies discussed in Section 3.2.1 demonstrate that reducing roughness improves bond formation. From a reliability perspective, the important result is that nonuniform surface morphology can produce local bonding failures even when the average surface roughness appears acceptable.

Film-transfer conditions can introduce an additional yield constraint. Yamamoto et al. [47] reported that direct transfer of continuous Au films reduced rough-surface RMS from 1.6 nm to 0.4 nm, while transferred films thinner than 5 nm increased the roughness. Higurashi et al. [48] later showed that repeated thin-film transfer reduced rough Au surfaces, and Au–Au bonding at 150 °C succeeded only after smoothing treatment. These findings indicate that an excessively thin or discontinuous transfer layer can reproduce the underlying roughness rather than conceal it, thereby reducing bonding yield.

Another yield limiter is the combination of Au film thickness and roughness in wafer-scale bonding. As shown in Section 3.2.1, ref. [49] found that bonded area and wafer-scale vacuum sealing both depended on staying below specific Au-thickness thresholds. This wafer-scale result shows that low roughness alone does not guarantee high yield; film thickness must also allow for sufficient conformity across the bonding area while maintaining the required sealing geometry.

A second major limiter is surface condition after activation. In surface-activated bonding, the activated Au surface does not remain highly bondable indefinitely after exposure to air. Takeuchi et al. [77,96] show that a self-assembled monolayer was used to protect the activated surface and extend the room-temperature bonding window. Activation therefore introduces a time-dependent process window: delays between activation and contact, together with uncontrolled exposure to air, can reduce yield unless the prepared surface is protected.

For processes that continue to use thermal and mechanical assistance, the limiting factors are different. For TCB, yield depends on applying sufficient temperature and pressure to disrupt surface barriers, deform asperities, and create real contact. Ang et al. [23] identified a critical temperature below which bonding did not occur, confirming that insufficient thermal input can produce complete bond failure rather than only reduced strength. Further, yield is also influenced by alignment and overlap [15,60,97]. Unlike nanoscale roughness and contamination, alignment is a device-scale limitation: misalignment or insufficient pad overlap reduces the available contact area and can produce open interconnects even when the opposing Au surfaces are individually bondable. The reviewed evidence therefore shows that bonding yield is governed by interacting surface-scale and device-scale factors rather than by only one process parameter.

### 6.2. Mechanical Integrity

Even when a high bonded-area yield is achieved, the resulting interface must withstand the mechanical loads associated with fabrication, packaging, and operation. The low-temperature SAB studies consistently show that adequate mechanical strength can be obtained at low temperature when the Au surfaces are sufficiently smooth and active. Representative VCSEL and wafer-bonding studies met device-level shear requirements without requiring conventional high-temperature bonding [55,59,63].

For planarized and transferred Au surfaces, several studies reported either fracture within the substrate or bonding strength comparable to or greater than the bulk-material fracture limit [41,43,46,47,49,98]. Such results indicate that the Au–Au interface was not the weakest part of the bonded structure.

By comparison, the TCB literature shows how mechanical strength changes with the applied process conditions. Ang et al. [23] showed that TCB strength depends on the combined effects of temperature and pressure. Bond strength increased as real contact developed, but tensile strength eventually reached a maximum with increasing pressure. This indicates that pressure does not improve joint strength indefinitely once sufficient interfacial contact has been established.

For flexible structures, a single strength measurement is not sufficient because the joint is exposed to repeated deformation. Takakuwa et al. [7] and Takamatsu et al. [76] combined peel testing with cyclic deformation. In the reported peel tests, failure occurred in the polymer substrate or device structure rather than through simple separation of the Au–Au interface, indicating that the bond remained mechanically robust despite the compliance of the surrounding materials.

In FHE, the key reliability question is whether the joint remains mechanically and electrically stable during repeated deformation. Takamatsu et al. [76] reported no noticeable sensor drift or degradation after 10,000 bending cycles. This cyclic evaluation is more representative of practical flexible-device operation than a single shear or peel measurement.

The mechanical literature therefore shows that initial strength, failure location, and resistance to repeated thermal or mechanical loading should be considered together.

### 6.3. Electrical Functionality and Stability

Mechanical survival must also be accompanied by stable electrical performance. Imamura et al. [59] showed that SAB-bonded VCSELs retained normal L-I-V behavior after bonding, indicating that the bonding process preserved optoelectronic functionality. Sorensen et al. [34] reported daisy-chain resistance values for InP dies bonded onto a silicon interconnect fabric; the interconnects remained intact after thermal cycling. Sharma et al. [18] also reported electrical continuity across all 21 daisy chains in their direct Au pillar thermocompression structures. A major limitation of the existing literature is the lack of systematic testing under sustained current, elevated temperature, and combined electrical–mechanical loading. Contact-resistance evolution, current-carrying capacity, electromigration, and failure under accelerated aging remain insufficiently characterized. As a result, many studies demonstrate successful electrical connection, but fewer establish the lifetime of the Au–Au interface under realistic operating conditions.

### 6.4. Stress-Specific Reliability and Benchmarking

Reliability should be evaluated from the initial bond quality and from its stability under relevant operating conditions. For rigid wafer and die structures, useful tests include thermal cycling or thermal shock, high-temperature storage, humidity exposure, and electrical current stressing when applicable. Bond strength, contact resistance, bonded area, and interface condition should be measured after testing. For flexible structures, bending radius, number of cycles, bending direction, and resistance change should also be reported. Hermetic packages should include leak-rate or cavity-pressure measurements before and after aging. Rather than using a single bond-strength value as a universal benchmark, reliability can be compared based on how well the mechanical, electrical, and hermetic properties are retained after a defined stress test. For cross-study comparison, 500 thermal cycles can serve as a practical reference benchmark for rigid Au–Au interconnects based on previous demonstrations [34] while 10,000 bending cycles can serve as a reference benchmark for flexible Au–Au structures [7,76]. These values should be treated as comparative reference points rather than universal qualification limits because the required reliability depends on the intended application.

For wafer-scale studies, the number and location of tested dies or bonding sites across the wafer, initial bonding yield, and yield after reliability testing should also be reported. The reviewed literature remains uneven in this respect. Thermal cycling, bending, and long-term sealing have been demonstrated in selected studies, while humidity exposure, sustained current stressing, electromigration, and combined thermal, electrical, and mechanical loading are less commonly reported. More consistent test conditions and reporting would make the reliability of different Au–Au bonding approaches easier to compare.

## 7. Research Gaps and Future Directions

The reviewed literature shows that low-temperature and room-temperature Au–Au bonding has moved beyond proof-of-concept demonstrations. Many studies have already shown strong bonding, electrical continuity, hermetic sealing, or device operation. The remaining challenge is to translate the surface requirements identified in this review into repeatable manufacturing processes. The key future goal is to define manufacturable processes for plated, patterned, rough, and non-planar Au surfaces in real device structures.

The first priority is to establish a practical and quantitative definition of surface readiness. Low-temperature bonding depends on the combined effects of surface roughness, contamination, activation state, and the delay between activation and contact. These factors are often studied separately, although they interact during the actual bonding process. Future studies should therefore determine acceptable combinations of roughness and activation state rather than reporting a single roughness threshold or activation treatment in isolation. They should also quantify how quickly Au surfaces lose activated bonding ability in air under different humidity, temperature, storage time, and handling conditions. Surface-protection methods, such as SAM-based protection or temporary passivation, should be evaluated using the same surface, bonding, and reliability metrics.

The second priority is wafer-scale control of rough and patterned Au. Smooth sputtered Au films can support room-temperature bonding, whereas many practical seal rings, bumps, and interconnects rely on electroplated Au with substantially higher roughness. Template stripping, imprint flattening, thin-film transfer, and mechanical planarization have shown strong potential, but their scalability depends on whether the required surface condition can be reproduced uniformly over the full wafer. At wafer scale, local roughness, plated-height variation, particle contamination, wafer bow, and nonuniform activation can produce spatial variations in real contact area and therefore in bond yield. Alignment accuracy also becomes increasingly important as pad pitch and bonding features decrease, because local misregistration reduces overlap and can generate open or partially bonded interconnects even when the individual Au surfaces are bondable. Surface preparation should therefore be evaluated not only by local AFM measurements but also by wafer-level distributions of roughness, activation state, particle density, and activation-to-contact delay. Future studies should report these spatial variations together with alignment error, bonded-area distribution, defect density, transfer yield, and sealing or electrical yield across full wafers. This is particularly important for 200 mm and 300 mm integration flows, where process uniformity rather than isolated local bond quality becomes the main manufacturability requirement.

The third priority is bonding on realistic patterned, rough, and non-planar Au surfaces. Many room-temperature bonding studies use relatively flat test structures, while practical devices may contain seal rings, microbumps, feedthroughs, cavities, and patterned electrodes. In these structures, low surface roughness alone may not be enough for successful bonding. Differences in feature height, local planarity, edge shape, and plated thickness can prevent full contact between the two Au surfaces. This is particularly important for electroplated Au, where both surface roughness and larger-scale height variations can affect bonding. Therefore, smoothing methods such as imprint flattening, thin-film transfer, template stripping, and mechanical planarization should be evaluated not only by Ra or RMS roughness, but also by how well they maintain feature shape and provide uniform contact. Future studies should also report feature height, planarity, bonded area, alignment, electrical continuity, leak performance when relevant, and mechanical strength.

The fourth priority is reducing bonding pressure without losing interface quality. High pressure can damage fragile devices, which is important for MEMS membranes, photonic chips, thin semiconductor dies, flexible sensors, and heterogeneous stacks with CTE mismatch. Future studies should examine how bump geometry and Au thickness control local deformation and contact formation. Important parameters include bump height, pitch, contact area, tip radius, sidewall geometry, and Au thickness. Finite-element modeling can support this work; however, the models should be validated using real bonded interfaces. The objective should be to determine how much bonding pressure is required for surfaces with different roughness and activation conditions, rather than optimizing pressure independently.

Reliability testing should become a central part of future Au–Au bonding studies. Many papers report initial bond strength, bonded area, or electrical continuity, but fewer studies examine long-term behavior under operating stress. Future work should include thermal cycling, high-temperature storage, damp-heat exposure, current stressing, and cyclic bending when relevant. Cross-sectional analysis after reliability testing is also important; it can show whether failure starts from the voids, cracks, grain-boundary changes, interfacial contamination, or adhesion loss. This would connect the initial surface-readiness condition directly to long-term interface stability.

The main future direction is therefore to move from demonstrating successful Au–Au bonding toward a validated surface-readiness framework. Such a framework should define how surface roughness and activation state interact with film thickness, feature geometry, bonding pressure, environmental exposure, and processing delay. It should then be translated into wafer-scale process windows and verified under application-specific reliability conditions. This transition is necessary for low-temperature Au–Au bonding to progress from laboratory feasibility to manufacturable device integration.

## 8. Conclusions

Au–Au bonding is a promising interconnection and sealing approach for heterogeneous integration, MEMS packaging, optoelectronic devices, sensors, and flexible electronics. Its main advantage is the ability to form a direct metallic interface with high electrical conductivity, good mechanical stability, and strong resistance to oxidation. The reviewed studies show that bonding success is mainly governed by the condition of the Au surfaces: surface roughness, cleanliness, activation method, pressure, temperature, and Au film structure all play critical roles.

Low-temperature and room-temperature Au–Au bonding have expanded direct metallic joining beyond conventional high-temperature packaging toward MEMS, optoelectronics, heterogeneous integration, wafer-level sealing, sensors, and flexible electronics. The reviewed evidence shows that reducing bonding temperature and pressure transfers greater importance to the condition of the Au surface before contact, as these factors jointly determine whether sufficient real contact can be achieved. TCB can compensate for some surface limitations through heat-assisted deformation and diffusion. The room-temperature SAB generally requires much stricter surface preparation. WVPAB and surface-smoothing approaches demonstrate possible routes for extending direct bonding to rougher and mechanically compliant structures.

Four challenges remain particularly important for manufacturing. First, surface readiness must be defined using measurable combinations of roughness, contamination, activation state, and activation-to-bond delay rather than by roughness alone. Second, smoothing and activation processes must be demonstrated uniformly over wafer-scale patterned and electroplated Au structures. Third, bonding mechanisms and process windows need to be validated for realistic bumps, seal rings, non-planar features, and reduced pressure joining. Fourth, reliability assessment must expand from initial shear strength and electrical continuity toward standardized thermal, humidity, electrical, hermetic, and mechanical aging tests. Addressing these issues would move low-temperature Au–Au bonding from successful laboratory demonstrations toward reproducible and manufacturable integration processes.

## Figures and Tables

**Table 1:** Key terminology used in this review.

Term	Meaning in This Review
Au–Au direct bonding	Umbrella term for joining Au surfaces without solder or polymer adhesive as the bonding medium.
Surface-activated bonding (SAB)	A direct-bonding route in which surfaces are activated before contact, typically by plasma, ion, or fast-atom treatment, to remove barriers and increase bondability at low or room temperature.
Thermocompression bonding (TCB)	A direct metal-bonding route relying primarily on simultaneous elevated temperature and compressive pressure to increase real contact and promote interface formation.
Hybrid bonding	Simultaneous bonding of metal contacts and surrounding dielectric surfaces; therefore broader than Au–Au metal direct bonding and not synonymous with SAB or TCB.

**Table 2:** Literature search strategy and screening results.

Item	Description
Database	Scopus and Web of Science
Search field	Scopus: Title, abstract, keywords; Web of Science: Topic (TS)
Scopus query	TITLE-ABS-KEY((“Au-Au bonding” OR “Au/Au bonding” OR “gold-gold bonding” OR “gold to gold bonding”) AND (“low-temperature” OR “room temperature” OR thermocompression OR “surface activated bonding” OR “surface-activated bonding” OR “plasma activated bonding” OR “water vapor plasma assisted bonding” OR WVPAB) AND (“heterogeneous integration” OR packaging OR wafer OR chip OR die OR “3D integration” OR “3D IC” OR flexible OR “flexible hybrid electronics” OR FHE))
Web of Science query	TS = ((“Au-Au bonding” OR “Au/Au bonding” OR “gold-gold bonding” OR “gold to gold bonding”) AND (“low-temperature” OR “room temperature” OR thermocompression OR “surface activated bonding” OR “surface-activated bonding” OR “plasma activated bonding” OR “water vapor plasma assisted bonding” OR WVPAB) AND (“heterogeneous integration” OR packaging OR wafer OR chip OR die OR “3D integration” OR “3D IC” OR flexible OR “flexible hybrid electronics” OR FHE))
Search date	17 June 2026

## Data Availability

No new data were created or analyzed in this study. Data sharing is not applicable to this article.

## Abbreviations

The following abbreviations are used in this manuscript:

Abbreviation	Full form
AFM	Atomic force microscopy
Ar	Argon
Au	Gold
CMOS	Complementary metal–oxide–semiconductor
CMP	Chemical mechanical polishing
CMUT	Capacitive micromachined ultrasonic transducer
CTE	Coefficient of thermal expansion
DDT	Dodecanethiol
EDX	Energy-dispersive X-ray spectroscopy
FAB	Fast atom beam
FBAR	Film bulk acoustic resonator
FHE	Flexible hybrid electronics
FIB	Focused ion beam
H_2_	Hydrogen
HI	Heterogeneous integration
IC	Integrated circuit
I/O	Input/output
L-I-V	Light-current-voltage
L/S	Line/space
LSI	Large-scale integrated circuit
LTCC	Low-temperature co-fired ceramic
MEMS	Microelectromechanical systems
MIL-STD	Military standard
N_2_	Nitrogen
NP	Nanoparticle
O_2_	Oxygen
OLED	Organic light-emitting diode
PI	Polyimide
PRISMA	Preferred Reporting Items for Systematic Reviews and Meta-Analyses
RF	Radio frequency
RF-MEMS	Radio-frequency microelectromechanical systems
RGP	Residual gas pressure
RMS	Root mean square
RT	Room temperature
SAB	Surface-activated bonding
SAM	Self-assembled monolayer
SAW	Surface acoustic wave
TCB	Thermocompression bonding
TEM	Transmission electron microscopy
TSV	Through-silicon via
UHV	Ultra-high vacuum
UV/O_3_	Ultraviolet/ozone
VCSEL	Vertical-cavity surface-emitting laser
VUV	Vacuum ultraviolet
WVPAB	Water-vapor plasma-assisted bonding
XPS	X-ray photoelectron spectroscopy

## Appendix A

**Table A1:** The metrology values reported for selected Au–Au bonding studies.

Study	Bonding Route	Bonding Temperature (°C)	Pressure/Load	Bonding Time	Au Thickness/Interface Stack	Activation/Pretreatment	Geometry	Surface Roughness	Bond Strength	Electrical/Hermetic/Reliability Outcome
[34]	TCB	=250	100–350 MPa	10–20 s	NR	NR	Au pillars = 5 × 5 × 5 µm^3^; InP die = 100 µm	Pillar RMS = 40 nm	38–238 MPa	≤1.5 Ω/die; 500 thermal cycles; 100% survived
[18]	TCB	350	220–250 N	250–500 s	Au pillars: 50 µm diameter, 200 µm pitch, 2–3 µm height	No specific surface-activation treatment reported; passivation-free TCB	21 × 20 array = 420 bonds	NR	Average shear strength = 128 MPa	All 21 daisy chains electrically connected
[38]	TCB + Si–Si	380	7000 mbar	1 h	Ti/Au = 40/200 nm	O_2_-plasma treatment used for Si–Si hydrophilic prebonding, not as Au-surface activation before Au–Au TCB	Package = 9.1 × 8.5 mm^2^; cavity = 9.0 ± 0.2 µm	NR	Average packaging strength = 26 MPa	Static capacitance yield > 77%; hermeticity yield > 90%
[58]	SAB	150	500 gf	30 s contact	VCSEL electrode: Ni 3 µm/Au 100 nm; substrate Au = 500 nm	Ar RF plasma, 100 W, 30 s	Electrode diameter = 50 µm	NR	Bond strength = 84 MPa	Normal VCSEL operation after bonding
[59]	SAB	25–200	50–900 gf	30 s	VCSEL electrode: Ni 3 µm/Au 100 nm; substrate Au = 500 nm	Ar RF plasma: 100 W, 0–180 s	NR	RMS = 5.6 nm before plasma and 7.0 nm after 30 s Ar plasma	Representative condition (150 °C, 500 gf, 30 s plasma): strength > 50 gf, corresponding to about 54 MPa	No L-I-V degradation
[63]	SAB	RT	Manual contact	-	Au < 50 nm	Ar RF plasma = 100 W, 30 s; contact within 5 min	NR	RMS < 0.5 nm; 15 nm Au: 0.24 nm; 75 nm Au: 1.14 nm	47–70 MPa	Atomic-scale TEM interface
[64]	SAB	RT	No compressive load	Manual contact	Au = 30 nm; Ti = 3 nm	Air exposure = 33–2000 h; Ar RF plasma = 30 s	NR	Rrms = 0.43 nm; plasma: 0.39–0.42 nm	48–72 MPa	Bonding front < 1 s; atomic-scale TEM after 2000 h
[67]	Plasma-SAB	RT	Manual contact	-	Au = 15 nm; Ti = 5 nm	Plasma = 30–120 s; anneal test = 150 °C, 10 min	NR	RMS = 0.38 nm; O_2_ contact angle < 5°	Ar: >2.5 J/m^2^/wafer fracture; O_2_: 0.1–0.2 J/m^2^; O_2_ + anneal: 2.5 J/m^2^	Au_2_O_3_ desorption = 110 °C
[41]	Imprint + SAB	Final bonding: room temperature; imprint = 200 °C	Imprint = 150 MPa; final bonding load = 40 kN	-	Electroplated Au around 10 µm thick; opposing Au film = 50 nm	Ar fast-atom-beam treatment	-	Sq 16 to 3 nm; Sz 113 to 53 nm	>200 MPa	NR
[48]	Transfer + SAB	150	Transfer = 200 MPa; final bonding = 75 MPa	600 s	Target rough Au = 200 nm on 20 nm Ti; transferred Au film = 300 nm; opposing smooth Au film = 200 nm	Ar/H_2_ atmospheric-pressure plasma; smoothing transfer: 150 °C, 200 MPa, 60 s	-	RMS: 205 to 10 nm over 90 × 90 µm^2^; RMS: 3.1 to 0.8 nm over 1 × 1 µm^2^	around 15 MPa (read from figure); bulk/substrate fracture	NR
[49]	SAB sealing	Room temperature	<1.6 MPa	-	Au = 15–500 nm; Ti = 5 nm	Ar-plasma activation = 60 s	AFM scan = 500 × 500 nm^2^	RMS = 0.3–1.6 nm	Bonded area > 85% for Au ≤ 100 nm	Vacuum sealing achieved for Au ≤ 50 nm
[99]	TCB sealing	300	40 MPa	30 min	Au signal line = 1 µm; SiOx = 2–12 µm	Activation: Ar plasma immediately before bonding; vacuum around 5 × 10^−3^ Pa	Planarized Au seal-ring surface	around 1 nm (Ra)	NR	Cavity pressure around 500 Pa or lower; vacuum maintained for >19 months; leak rate < 8 × 10^−16^ Pa·m^3^/s
[7]	WVPAB	RT	=2 N for 5 s; no sustained load	≥12 h after overlap	2 µm parylene; Cr/Au = 3.5/50–100 nm	Water-vapor plasma = 50 W, 40 s	NR	RMS = 6.29 nm; smooth Au RMS = 1.8 nm	Substrate failure in peel test	Contact area < 50 × 50 µm^2^; L/S = 10 µm; <1% ΔR after 10,000 cycles
[53]	Getter-layer SAB	Room temperature	Bonding pressure = 123 kPa	-	Getter layer: Au/Ti/Au = 20/100/20 nm; opposing bonding layer: Au/Ti = 20/5 nm	Ar plasma = 200 W, 30 s; vacuum = 1 × 10^−2^ Pa	NR	RMS = 3.48 to 0.538 nm	Strength > 26 MPa	Unbonded area = 9.5%
[19]	Au–Au thermal interface	300	Pressure = 7–11 MPa; optimum = 9 MPa	30 min (Methods; abstract states 1 h)	Ti/Ni = 20/20 nm; Au thickness = 100, 300, and 500 nm	No CMP and no post-anneal (ICP substrate treatment not classified as Au surface activation)	NR	RMS = 1.18, 1.77, and 2.29 nm, respectively	Mechanical bond strength = NR	500 nm Au gave 99% bonding ratio; thermal conductivity = 101.32 W·m^−1^·K^−1^
[50]	PI/SiO_2_ template stripping + SAB	Smoothing = 150 °C; final bonding = 30 °C	Smoothing = 75 MPa; final bonding = 30 MPa	600 s	Plated Au = 1.25 µm; template Au = 100 nm; opposing Au = 15 nm	Ar RF plasma	-	RMS = 21 nm to around 6 nm after PI transfers to around 1 nm after additional SiO_2_/Si transfer	Strength not assigned an exact MPa value (primarily read from plotted data); MIL-STD-883 criterion satisfied; Si bulk fracture	NR
[52]	PI template stripping + SAB	Smoothing = 150 °C; final bonding = 30 °C	Smoothing = 75 MPa; final bonding = 10 MPa	10 min	Plated Au = 1.2 µm; template Au = 100 nm; opposing Au = 15 nm	Smoothing activation: Ar RF plasma, 200 W, 1 min; final activation: Ar plasma, 150 W, 30 s	-	RMS around 21.3 to around 5.3 nm over 10 × 10 µm^2^ after repeated PI template stripping	Strength > 15 MPa with bulk fracture	NR
[51]	Bump smoothing + SAB	Smoothing = 150 °C; final bonding = 30 °C	Smoothing = 75 MPa/bump; final bonding = 30 MPa/bump	10 min in air	Template Au = 100 nm; opposing Au = 15 nm	Smoothing activation = Ar fast atom beam for 5 min	Au bumps = 100 µm diameters, around 2.5 µm height, 400–500 µm pitch	RMS = around 30 to around 6 nm after three PI transfers, 10 × 10 µm^2^ AFM area	Bond strength = NR, because the paper does not report a quantitative final strength	NR
[76]	WVPAB flexible device	RT; post-anneal = 200	Hand press; force NR	-	Si sensor = 5 µm; parylene = 2 µm; piezoresistor = 150 nm	Water-vapor plasma = 50 W, 40 s; post-anneal = 1 h	-	Au pad RMS = 1.4 nm; Au wiring RMS = 0.4 nm	Peel: sensor/substrate failure	10,000 bending cycles; no noticeable drift/degradation; sensitivity = 0.0712 per mm^−1^ curvature
[100]	TCB	150–175	250–450 N	10–20 min	NR	NR	Au pillars: 25 µm diameter, 100 µm pitch, 3–5 µm height	Ra = 5–10 nm	444.53 MPa at 150 °C; 573.91 MPa at 175 °C; cohesive Au failure	17 daisy chains showed electrical continuity.
[94]	Room-temperature direct bonding/cold welding	RT/low-T	Pressure investigated from 40 kPa to 10 MPa (10 MPa could damage the nanomesh); robust bonding around 1 MPa	Sufficient bond formation within around 3 s (pressing 10 s)	NR	No surface activation and no adhesive	Au-coated nanomesh interface	NR	T-peel strength around 10–20 N/m	Resistance change remained within 5% after 8000 bending cycles at 1 mm radius; EDX showed no C/O segregation

## References

[1] Huang M., Xia Y., Han X., Gao Y., Chen S., Cui Z., Zhang C., Gao Y., Qiao Z., Lv Y. (2025). High-strength Au–Au bonding for temperature and pressure integrated sensor. J. Mater. Res. Technol..

[2] Ren Z., Xu J., Le X., Lee C. (2021). Heterogeneous wafer bonding technology and thin-film transfer technology-enabling platform for the next generation applications beyond 5g. Micromachines.

[3] Tong Q.-Y., Gösele U. (1999). Semiconductor Wafer Bonding: Science and Technology.

[4] Hu H.W., Chen K.N. (2021). Development of low temperature Cu-Cu bonding and hybrid bonding for three-dimensional integrated circuits (3D IC). Microelectron. Reliab..

[5] Ko C.T., Chen K.N. (2012). Low temperature bonding technology for 3D integration. Microelectron. Reliab..

[6] Plößl A., Kräuter G. (1999). Wafer direct bonding: Tailoring adhesion between brittle materials. Mater. Sci. Eng. R Rep..

[7] Takakuwa M., Fukuda K., Yokota T., Inoue D., Hashizume D., Umezu S., Someya T. (2021). Direct gold bonding for flexible integrated electronics. Sci. Adv..

[8] Lau J.H. (2021). State-of-the-Art and Outlooks of Chiplets Heterogeneous Integration and Hybrid Bonding. J. Microelectron. Electron. Packag..

[9] Chidambaram V., Ren Q., Kawano M. (2019). Wafer level fusion and hybrid bonding: Impact of critical process parameters on bond quality. 2019 IEEE 21st Electronics Packaging Technology Conference (EPTC).

[10] Hsiung C.K., Chen K.N. (2024). A Review on Hybrid Bonding Interconnection and Its Characterization. IEEE Nanotechnol. Mag..

[11] Gao G., Workman T., Uzoh C., Bang K.M., Mirkarimi L., Theil J., Suwito D., Katkar R., Fountain G., Guevara G. (2020). Die to Wafer Stacking with Low Temperature Hybrid Bonding. 2020 IEEE 70th Electronic Components and Technology Conference (ECTC).

[12] Xu Y., Zeng Y., Zhao Y., Lee C., He M., Liu Z. (2025). A Review of Mechanism and Technology of Hybrid Bonding. J. Electron. Packag..

[13] Suga T., Itoh T., Xu Z., Tomita M., Yamauchi A. (2002). Surface activated bonding for new flip chip and bumpless interconnect systems. 52nd Electronic Components and Technology Conference 2002. (Cat. No.02CH37345).

[14] Rantamaki A., Lindfors J., Silvennoinen M., Kontio J., Tavast M., Okhotnikov O.G. (2013). Low temperature gold-to-gold bonded semiconductor disk laser. IEEE Photonics Technol. Lett..

[15] Takegawa Y., Kamakura M., Baba T., Okudo T., Taura T., Tomoida R., Shiroishi H., Tone K., Saijo T. 3-D wafer-level packaging of MEMS using surface activated bonding and through-Si vias. Proceedings of the IDW ‘07—Proceedings of the 14th International Display Workshops.

[16] Sinha K., Farley D., Kahnert T., Dasgupta A., Caers J.F., Zhao X.J. (2011). Au-Au ‘cold-weld’ bond strength in adhesively bonded flip-chip interconnects. 2011 12th Int. Conf. on Thermal, Mechanical and Multi-Physics Simulation and Experiments in Microelectronics and Microsystems.

[17] Sinha K., Farley D., Kahnert T., Solares S.D., Dasgupta A., Caers J.F., Zhao X.J. (2014). Influence of fabrication parameters on bond strength of adhesively bonded flip-chip interconnects. J. Adhes. Sci. Technol..

[18] Sharma N.K., Priya A., Sen P. (2025). High-Density Direct Au-Au Interconnects Using Thermo-Compression Bonding for Heterogeneous Integration. 2025 23rd International Conference on Solid-State Sensors, Actuators and Microsystems (Transducers).

[19] Shou T., Cheng L., Zhang C., Guo Y. (2024). Advanced Au-Au Direct Bonding for Enhanced Thermal Management in Heterogeneous Integration. 2024 25th International Conference on Electronic Packaging Technology (ICEPT).

[20] Vigna B., Ferrarini P., Villa F.F., Lasalandra E., Zerbini S. (2022). Silicon Sensors and Actuators: The Feynman Roadmap.

[21] Cirulis I., Kalangi D.S., Dubey V., Franz M., Wünsch D., Braun S., Haase M., Wiemer M., Schulz S.E. (2024). Fine Pitch Aluminum Hybrid Bonding: Overcoming the Challenges in Plating, Passivation and Bonding. 2024 IEEE 10th Electronics System-Integration Technology Conference (ESTC).

[22] Ang X.F., Zhang G.G., Tan B.K., Wei J., Chen Z., Wong C.C. (2005). Direct metal to metal bonding for microsystems interconnections and integration. 2005 7th Electronics Packaging Technology Conference.

[23] Ang X.F., Zhang G.G., Wei J., Chen Z., Wong C.C. (2006). Temperature and pressure dependence in thermocompression gold stud bonding. Thin Solid Films.

[24] Ang X.F., Li F.Y., Tan W.L., Chen Z., Wong C.C., Wei J. (2007). Self-assembled monolayers for reduced temperature direct metal thermocompression bonding. Appl. Phys. Lett..

[25] Chin L.C., Ang X.F., Wei J., Chen Z., Wong C.C. (2006). Enhancing direct metal bonding with self-assembled monolayers. Thin Solid Films.

[26] Unami N., Sakuma K., Mizuno J., Shoji S. (2010). Effects of excimer irradiation treatment on thermocompression Au-Au bonding. Jpn. J. Appl. Phys..

[27] Goorsky M.S., Schjølberg-Henriksen K., Beekley B., Bai T., Mani K., Ambhore P., Bajwa A., Malik N., Iyer S.S. (2018). Characterization of interfacial morphology of low temperature, low pressure Au-Au thermocompression bonding. Jpn. J. Appl. Phys..

[28] Park G.S., Yu J.H., Seo S.W., Choi W.B., Paek K.K., Sung M.Y., Park H.W., Yun S.K., Ju B.K. (2006). Wafer Level Hermetic Packaging for RF-MEMS Devices Using Electroplated Gold Layers. Key Eng. Mater..

[29] Zoumpoulidis T., Delft J., Wild M., Kuijpers P.E., Graaf P., Mauczok R., Biju K., Bartek M., Klee M., Dekker R. (2008). Ultrasonic and thermo-compression gold-to-gold bonding of narrow frames for hermetic cavity sealing and electrical interconnect. 2008 33rd IEEE/CPMT International Electronics Manufacturing Technology Conference (IEMT).

[30] Schjølberg-Henriksen K., Malik N., Sandvand A., Kittilsland G., Moe S.T. (2014). Leak Rates and Residual Gas Pressure in Cavities Sealed by Metal Thermo-Compression Bonding and Silicon Direct Bonding. ECS Trans..

[31] Tofteberg H.R., Schjølberg-Henriksen K., Fasting E.J., Moen A.S., Taklo M.M., Poppe E.U., Simensen C.J. (2014). Wafer-level Au-Au bonding in the 350-450 °C temperature range. J. Micromech. Microeng..

[32] Li Y., Guo X., Guan B., Chuai D., Shen G. (2009). High brightness ODR LED based on Au/Au wafer bonding. Guti Dianzixue Yanjiu Yu Jinzhan/Res. Prog. Solid State Electron..

[33] Karbownik P., Trajnerowicz A., Szerling A., Wójcik-Jedlińska A., Wasiak M., Pruszyńska-Karbownik E., Kosiel K., Gronowska I., Sarzała R., Bugajski M. (2015). Direct Au–Au bonding technology for high performance GaAs/AlGaAs quantum cascade lasers. Opt. Quantum Electron..

[34] Sorensen E., Vaisband B., Jangam S.C., Shirley T., Iyer S.S. (2019). Integration and characterization of InP die on silicon interconnect fabric. 2019 IEEE 69th Electronic Components and Technology Conference (ECTC).

[35] Yang Y.T., Hu C., Green J., Zhang P., Shakoorzadeh N., Ambhore P., Mogera U., Ni N., Wang K.L., Iyer S.S. (2020). Demonstration of Superconducting Interconnects on the Silicon Interconnect Fabric Using Thermocompression Bonding. 2020 IEEE 70th Electronic Components and Technology Conference (ECTC).

[36] Wang F., Dai J., Cui D., Kong Y. (2021). Low Temperature Wafer Level Au-Au Bonding for Heterogeneous Integration. 2021 IEEE MTT-S International Microwave Workshop Series on Advanced Materials and Processes for RF and THz Applications (IMWS-AMP).

[37] Bargiel S., Cogan J., Queste S., Oliveri S., Gauthier-Manuel L., Raschetti M., Leroy O., Beurthey S., Perrin-Terrin M. (2023). Comparison of Anodic and Au-Au Thermocompression Si-Wafer Bonding Methods for High-Pressure Microcooling Devices. Micromachines.

[38] Zhao K., Chen T., Wu W., Hu C., Liu H., Fan J. (2025). Sandwich Structure MEMS Accelerometer Packaging via Wafer-Level Tri-Layer Simultaneous Bonding. IEEE Sens. J..

[39] Takagi H., Maeda R., Chung T.R., Hosoda N., Suga T. (1998). Effect of surface roughness on room-temperature wafer bonding by Ar beam surface activation. Jpn. J. Appl. Phys..

[40] Goto S., Takeuchi K., Thu L.H.H., Matsumae T., Takagi H., Kurashima Y., Higurashi E. (2025). Au hollow pyramidal bumps for low-temperature low-pressure bonding fabricated via Au film transfer method. Sens. Actuators A Phys..

[41] Kurashima Y., Maeda A., Lu J., Zhang L., Takagi H. (2014). Room-temperature direct bonding of electroplated Au patterns flattened by a thermal imprint process. Microelectron. Eng..

[42] Kurashima Y., Maeda A., Takagi H. (2014). Replication of atomically smooth surface shape onto electroplated Au patterns by lift-off process and room-temperature Au-Au bonding in atmospheric air. Microelectron. Eng..

[43] Kurashima Y., Maeda A., Takagi H. (2015). Room-temperature bonding for hermetic sealing using electroplated Au seal rings with ultra-smooth surface. 2015 International Conference on Electronic Packaging and iMAPS All Asia Conference (ICEP-IAAC).

[44] Kurashima Y., Maeda A., Takagi H. (2016). Room-temperature wafer scale bonding using smoothed Au seal ring surfaces for hermetic sealing. Jpn. J. Appl. Phys..

[45] Kurashima Y., Matsumae T., Takagi H. (2018). Room-temperature Au–Au bonding in atmospheric air using direct transferred atomically smooth Au film on electroplated patterns. Microelectron. Eng..

[46] Kurashima Y., Maeda A., Takagi H. (2017). Direct transfer of atomically smooth Au film onto electroplated patterns for room-temperature Au-Au bonding in atmospheric air. 2017 5th International Workshop on Low Temperature Bonding for 3D Integration (LTB-3D).

[47] Yamamoto M., Matsumae T., Kurashima Y., Takagi H., Suga T., Itoh T., Higurashi E. (2019). Growth behavior of au films on SiO_2_ film and direct transfer for smoothing au surfaces. Int. J. Autom. Technol..

[48] Higurashi E., Yamamoto M., Nishimura R., Matsumae T., Kurashima Y., Takagi H., Suga T., Itoh T. (2020). Formation of smooth Au surfaces produced by multiple thin-film transfer process based on template stripping for low-temperature bonding. 2020 IEEE 70th Electronic Components and Technology Conference (ECTC).

[49] Yamamoto M., Matsumae T., Kurashima Y., Takagi H., Suga T., Takamatsu S., Itoh T., Higurashi E. (2020). Effect of Au film thickness and surface roughness on room-temperature wafer bonding and wafer-scale vacuum sealing by Au-Au surface activated bonding. Micromachines.

[50] Koseki S., Ogino M., Takeuchi K., Thu L.H.H., Matsumae T., Takagi H., Kurashima Y., Tsuda T., Tokuhisa T., Shimizu T. (2024). Template Stripping Process Combined with Polyimide and SiO_2_/Si Templates for Obtaining Smooth Au Surfaces. 2024 International Conference on Electronics Packaging (ICEP).

[51] Koseki S., Takeuchi K., Huong Thu L.H., Matsumae T., Takagi H., Kurashima Y., Tsuda T., Tokuhisa T., Shimizu T., Higurashi E. (2025). Smoothing of Plated Au Bumps Based on Template-Stripping for Low-Temperature Bonding. 2025 International Conference on Electronics Packaging and iMAPS All Asia Conference (ICEP-IAAC).

[52] Takeuchi K., Koseki S., Thu L.H.H., Matsumae T., Takagi H., Kurashima Y., Tsuda T., Tokuhisa T., Shimizu T., Higurashi E. (2025). Room temperature bonding of Au plating through surface smoothing using polyimide template stripping. Sens. Actuators A Phys..

[53] Matsumae T., Kariya S., Kurashima Y., Thu L.H.H., Higurashi E., Hayase M., Takagi H. (2022). Wafer-Scale Room-Temperature Bonding of Smooth Au/Ti-Based Getter Layer for Vacuum Packaging. Sensors.

[54] Hirano H., Hikichi K., Tanaka S. (2015). The wafer-level vacuum sealing and electrical interconnection using electroplated gold bumps planarized by single-point diamond fly cutting. 2015 Transducers—2015 18th International Conference on Solid-State Sensors, Actuators and Microsystems (TRANSDUCERS).

[55] Higurashi E., Suga T., Sawada R. (2005). Low-temperature direct flip-chip bonding for integrated micro-systems. 2005 IEEE LEOS Annual Meeting Conference Proceedings.

[56] Takigawa R., Higurashi E., Suga T., Shinada S., Kawanishi T. Low temperature bonding of LiNbO_3_ waveguide chips to Si substrates in air. Proceedings of the Optomechatronic Micro/Nano Devices and Components.

[57] Takigawa R., Higurashi E., Suga T., Shinada S., Kawanishi T. Low-temperature bonding of a LiNbO_3_ waveguide chip to a Si substrate in ambient air for hybrid-integrated optical devices. Proceedings of the Optomechatronic Micro/Nano Devices and Components II.

[58] Imamura T., Higurashi E., Suga T., Sawada R. (2006). Low temperature direct bonding of flip-chip mounting VCSEL to Si substrate. 2006 International Microsystems, Package, Assembly Conference Taiwan.

[59] Imamura T., Higurashi E., Suga T., Sawada R. (2008). Low-temperature direct bonding of flip-chip mountable VCSELs with Au-Au surface activation. IEEJ Trans. Sens. Micromachines.

[60] Takigawa R., Higurashi E., Suga T., Kawanishi T. (2011). Passive alignment and mounting of LiNbO_3_ waveguide chips on Si substrates by low-temperature solid-state bonding of Au. IEEE J. Sel. Top. Quantum Electron..

[61] Takigawa R., Higurashi E., Suga T., Sawada R. (2008). Room-temperature bonding of vertical-cavity surface-emitting laser chips on Si substrates using Au microbumps in ambient air. Appl. Phys. Express.

[62] Kunimune Y., Okumura K., Higurashi E., Suga T., Hagiwara K. (2016). Room-temperature wafer bonding using smooth gold thin films for wafer-level MEMS packaging. 2016 International Conference on Electronics Packaging (ICEP).

[63] Higurashi E., Okumura K., Kunimune Y., Suga T., Hagiwara K. (2017). Room-temperature bonding of wafers with smooth au thin films in ambient air using a surface-activated bonding method. IEICE Trans. Electron..

[64] Okumura K., Higurashi E., Suga T., Hagiwara K. (2015). Influence of air exposure time on bonding strength in Au-Au surface activated wafer bonding. 2015 International Conference on Electronic Packaging and iMAPS All Asia Conference (ICEP-IAAC).

[65] Yamamoto M., Matsumae T., Kurashima Y., Takagi H., Suga T., Itoh T., Higurashi E. (2018). Room-Temperature Wafer Bonding Using Smooth Au Thin Films for Integrated Plasmonic Devices. 2018 International Conference on Optical MEMS and Nanophotonics (OMN).

[66] Yamamoto M., Matsumae T., Kurashima Y., Takagi H., Suga T., Itoh T., Higurashi E. (2018). Surface analysis of argon and oxygen plasma-treated gold for room temperature wafer scale gold-gold bonding. 2018 IEEE CPMT Symposium Japan (ICSJ).

[67] Yamamoto M., Matsumae T., Kurashima Y., Takagi H., Suga T., Itoh T., Higurashi E. (2019). Investigation of plasma treatment conditions for wafer-scale room-temperature bonding using ultrathin Au films in ambient air. IEEJ Trans. Sens. Micromach..

[68] Ogino M., Takeuchi K., Higurashi E. (2024). Influence of Various Plasma and UV/O3Treatments on Au Surfaces for Au-Au Surface Activated Bonding. 2024 International Conference on Electronics Packaging (ICEP).

[69] Higurashi E., Yamamoto M., Ikeda S., Suga T., Sawada R. (2014). Low-temperature gold-gold bonding using argon and hydrogen gas mixture atmospheric-pressure plasma treatment for optical microsystems. 2014 International Conference on Optical MEMS and Nanophotonics (OMN).

[70] Higurashi E., Yamamoto M., Sato T., Suga T., Sawada R. (2016). Room-temperature gold-gold bonding method based on argon and hydrogen gas mixture atmospheric-pressure plasma treatment for optoelectronic device integration. IEICE Trans. Electron..

[71] Yamamoto M., Matsumae T., Kurashima Y., Takagi H., Miyake T., Suga T., Itoh T., Higurashi E. (2019). Wafer-scale Au-Au surface activated bonding using atmospheric-pressure plasma. 2019 International Conference on Electronics Packaging (ICEP).

[72] Matsuoka S., Yamamoto M., Higurashi E., Suga T., Sawada R. (2015). Low temperature Au-Au surface-activated bonding using nitrogen atmospheric-pressure plasma treatment for optical microsystems. 2015 International Conference on Electronic Packaging and iMAPS All Asia Conference (ICEP-IAAC).

[73] Okada A., Nimura M., Unami N., Shigetou A., Noma H., Sakuma K., Shoji S., Mizuno J. (2012). Low temperature Au-Au flip chip bonding with VUV/O_3_ treatment for 3D integration. 2012 3rd IEEE International Workshop on Low Temperature Bonding for 3D Integration (LTB-3D).

[74] Ogino M., Takeuchi K., Higurashi E. (2024). Sequential Surface Treatment Process with VUV light and Ar Plasma for Room Temperature Au-Au Bonding. 2024 IEEE CPMT Symposium Japan (ICSJ).

[75] Ogino M., Takeuchi K., Higurashi E. (2025). Investigation of Surface Activation Conditions Using VUV Light to Enable Room-Temperature Au–Au Bonding. 2025 IEEE CPMT Symposium Japan (ICSJ).

[76] Takamatsu S., Takakuwa M., Fukuda K., Yamamoto M., Tomita N., Liu H., Kobayashi T., Yokota T., Someya T., Itoh T. (2025). Flexible no-drift data glove using ultrathin silicon for the metaverse. Device.

[77] Takeuchi K., Wang J., Suga T., Kim B., Higurashi E. (2023). Protection of Activated Au Surface using Self-assembled Monolayer for Room Temperature Bonding. 2023 International Conference on Electronics Packaging (ICEP).

[78] Fang J.P., Cai J., Wang Q., Geng Z.T. (2020). Low Temperature Au-Au Bonding Using Ag Nanoparticles as Intermediate. 2020 IEEE 70th Electronic Components and Technology Conference (ECTC).

[79] Fang J.P., Cai J., Wang Q., Zheng K., Zhou Y.K., Geng Z.T. (2022). Low temperature Au-Au bonding using Ag nanoparticles as intermediate for die attachment in power device packaging. Appl. Surf. Sci..

[80] Higurashi E., Suga T. (2016). Review of Low-Temperature Bonding Technologies and Their Application in Optoelectronic Devices. Electron. Commun. Jpn..

[81] Okumura K., Higurashi E., Suga T., Hagiwara K. (2014). Low-temperature GaAs/SiC wafer bonding with Au thin film for high-power semiconductor lasers. 2014 International Conference on Electronics Packaging (ICEP).

[82] Takeuchi K., Higurashi E. (2025). Wafer Bonding of GaAs and SiC via Thin Au Film at Room Temperature. Micromachines.

[83] Usui M., Takeda K., Hirata H., Fukuda H., Tsuchizawa T., Nishi H., Kou R., Hiraki T., Honda K., Nogawa M. (2015). Opto-electronic hybrid integrated chip packaging technology for silicon photonic platform using gold-stud bump bonding. 2015 International Conference on Electronic Packaging and iMAPS All Asia Conference (ICEP-IAAC).

[84] Yamada S., Shim C.H., Edura T., Okada A., Adachi C., Shoji S., Mizuno J. (2016). Fabrication of bottom-emitting organic light-emitting diode panels interconnected with encapsulation substrate by Au-Au flip-chip bonding and capillary-driven filling process. Microelectron. Eng..

[85] Zoschke K., Manier C.A., Wilke M., Jurgensen N., Oppermann H., Ruffieux D., Dekker J., Heikkinen H., Piazza S.D., Allegato G. (2013). Hermetic wafer level packaging of MEMS components using through silicon via and wafer to wafer bonding technologies. 2013 IEEE 63rd Electronic Components and Technology Conference (ECTC).

[86] Matsumae T., Yuichi K., Higurashi E., Takagi H. (2018). Surface activated bonding of Au/Ta layers after degas annealing for MEMS packaging. 2018 International Conference on Electronics Packaging and iMAPS All Asia Conference (ICEP-IAAC).

[87] Matsumae T., Kurashima Y., Takagi H. (2018). Surface activated bonding of Ti/Au and Ti/Pt/Au films after vacuum annealing for MEMS packaging. Microelectron. Eng..

[88] Yildiz F., Matsunaga T., Haga Y. (2018). Fabrication and packaging of CMUT using low temperature co-fired ceramic. Micromachines.

[89] Ballandras S., Courjon E., Baron T., Moulet J.B., Signamarcheix T., Daniau W. (2015). LiTaO_3_/silicon composite wafers for the fabrication of low loss low TCF high coupling resonators for filter applications. Phys. Procedia.

[90] Park K., Esashi M., Tanaka S. (2010). Wafer-level heterointegration process of SAW devices and LSI. 2010 IEEE Ultrasonics Symposium (IUS).

[91] Park K.D., Esashi M., Tanaka S. (2011). Lithium-niobate-based surface acoustic wave device directly integrated on IC. 2011 IEEE International Ultrasonics Symposium (IUS).

[92] Tanaka S., Park K., Esashi M. (2012). Lithium-niobate-based surface acoustic wave oscillator directly integrated with CMOS sustaining amplifier. IEEE Trans. Ultrason. Ferroelectr. Freq. Control.

[93] Hikichi K., Seiyama K., Ueda M., Taniguchi S., Hashimoto K.Y., Esashi M., Tanaka S. (2014). Wafer-level selective transfer method for FBAR-LSI Integration. 2014 IEEE International Frequency Control Symposium (FCS).

[94] Ebihara Y., Takakuwa M., Yamagishi K., Yokota T., Someya T. (2026). Direct Bonding of Gold Nanomeshes at Low Temperature for Flexible and Breathable Electronics. ACS Appl. Mater. Interfaces.

[95] Ahn J.Y., Kim J.Y., Kim Y.S., Moon J.G., Kwon O.G., Park K.Y., Lee S.Y., Youm K.S., Jeong J.W., Ahn E.C. (2020). Reliability Study of Direct Au (Au-Cu plating) Surface Treatment on PKG Substrate. 2020 IEEE 22nd Electronics Packaging Technology Conference (EPTC).

[96] Takeuchi K., Wang J., Kim B., Suga T., Higurashi E. (2023). Room temperature bonding of Au assisted by self-assembled monolayer. Appl. Phys. Lett..

[97] Jeon G., Jeong M., Lee S., Jo Y., Kim N.S. (2025). Resonator Width Optimization for Enhanced Performance and Bonding Reliability in Wideband RF MEMS Filter. Micromachines.

[98] Kurashima Y., Maeda A., Takagi H. (2014). Room temperature wafer scale bonding of electroplated Au patterns processed by surface planarization. 2014 International Conference on Electronics Packaging (ICEP).

[99] Moriyama M., Suzuki Y., Totsu K., Hirano H., Tanaka S. (2019). Metal-bonding-based hermetic wafer-level MEMS packaging technology using in-plane feedthrough: Hermeticity and high frequency characteristics of thick gold film feedthrough. Electr. Eng. Jpn..

[100] Sharma N.K., Priya A., Sen P. (2025). Sub-200 °C Gold-Gold Bonding for Reliable 3D Heterogeneous Integration. 2025 IEEE 27th Electronics Packaging Technology Conference (EPTC).

